# Valorization of Berry Fruit By-Products: Bioactive Compounds, Extraction, Health Benefits, Encapsulation and Food Applications

**DOI:** 10.3390/foods14081354

**Published:** 2025-04-15

**Authors:** Sandra Pedisić, Zoran Zorić, Maja Repajić, Branka Levaj, Ana Dobrinčić, Sandra Balbino, Zrinka Čošić, Verica Dragović-Uzelac, Ivona Elez Garofulić

**Affiliations:** 1 Centre for Food Technology and Biotechnology, University of Zagreb Faculty of Food Technology and Biotechnology, P. Kasandrića 3, 23000 Zadar, Croatia; zrinka.cosic12@gmail.com; 2 Department of Ecology, Agronomy and Aquaculture, University of Zadar, Trg kneza Višeslava 9, 23000 Zadar, Croatia; zzoric@unizd.hr; 3 Faculty of Food Technology and Biotechnology, University of Zagreb, Pierottijeva 6, 10000 Zagreb, Croatia; maja.repajic@pbf.unizg.hr (M.R.); blevaj@pbf.hr (B.L.); ana.dobrincic@pbf.unizg.hr (A.D.); sandra.balbino@pbf.unizg.hr (S.B.); vdragov@pbf.hr (V.D.-U.); ivona.elez@pbf.unizg.hr (I.E.G.)

**Keywords:** berry by-products, bioactive compounds, extraction techniques, encapsulation, high value-added products

## Abstract

The increased production of high-quality berry products in recent years has led to considerable quantities of by-products such as pomace (25–50%), which consists of skin, seeds, stems and leaves. The improper management of pomace can lead to environmental pollution and potential public health problems due to microbial contamination, and storage causes additional processing costs. However, due to their high content of various valuable bioactive compounds (BACs), berry by-products have gained much attention as sustainable and functional ingredients with applications in the food and nutraceutical industries. The health benefits are primarily attributed to the phenolic compounds, which exhibit numerous biological activities, especially good antioxidant and antibacterial activity as well as health-promoting effects. This review summarizes the bioactive content and composition of extracts from berry by-products (genera Ribes, Rubus, Fragaria, Sambucus, Aronia and Vaccinium) obtained using advanced extraction technologies and their stabilization through sophisticated encapsulation technologies that make them suitable for various food applications. The addition of berry pomace to beverages, bakery, dairy and meat products improves sensory quality, extends shelf life, increases nutritional value and reduces the environmental footprint. This information can provide food scientists with valuable insights to evaluate the potential of berry by-products as functional ingredients with health-promoting and disease-preventing properties that create value-added products for human consumption while reducing food waste.

## 1. Introduction

Berries are an important fresh produce in Europe, both in terms of production volume and economic importance. According to Eurostat, the EU produced 700,000 tonnes of berries in 2023, with projections indicating that European berry production will reach approximately 1,020,000 tonnes by 2028 [1]. The most commonly consumed berry varieties include blueberries (*Vaccinium myrtillus*), raspberries (*Rubus idaeum*), blackberries (*Rubus fruticosus*), chokeberries (*Aronia melanocarpa*), strawberries (*Fragaria ananassa*), white currants *(Ribes niveum*), black currants (*Ribes nigrum*), and red currants (*Ribes rubrum*) [2]. Small berries are recognized worldwide as health-promoting fruits due to their high contents of phytochemicals and their proven numerous biological activities to reduce the risk of many chronic diseases [3]. However, fresh berries have a limited shelf life, and large quantities are usually processed into juice. This process generates substantial by-products, including pomace (25–35%), which consists of skins, seeds, and stems [4]. Pomace also contains various bioactive compounds (BACs), especially polyphenols with high antioxidant activity [5,6]. Currently, fruit pomace is either used as animal feed or discarded, which contributes to environmental pollution and limits its potential, as it is easily microbiologically contaminated and perishable due to its high moisture content (≈80%), so the possible storage increases processing costs [7]. Drying the berry pomace after juice pressing reduces the potential risk and extends its shelf life. Recovering berry by-products (BBPs) from the processing industry is crucial for economic growth, food security, and environmental sustainability. It also opens new opportunities for high-value-added products in the food, pharmaceutical, cosmetic, and sustainable packaging industries [8]. For example, the study by May and Guenther [9] demonstrated that optimizing blackcurrant juice by-product utilization could increase profits by up to 13%. The peel and seeds of berry pomace are rich in polyphenols, anthocyanins and dietary fiber and are less contaminated with pesticides, making them suitable for use in the functional food industry (baked goods, cereal-based snacks or smoothies) [9]. Berry leaves are also a potentially valuable source of BACs, which is a relatively new trend in the food industry. Traditionally, berry leaves have been used in herbal medicine to treat cold, inflammation, diabetes, and eye dysfunctions [10]. Maximizing the potential of BBPs relies on conventional and advanced extraction techniques—including ultrasound-assisted, microwave-assisted, pressurized liquid, high-pressure, and supercritical fluid extraction—to obtain high-quality extracts and isolate BACs [11]. An ideal extraction technique should efficiently extract target BACs from the plant matrix while offering high selectivity, reproducibility and suitability for analytical procedures and bioassays [12]. The choice of extraction technique also affects the cost, duration and accessibility of the procedure. However, the obtained extracts are often susceptible to oxidation and not stable under processing and storage conditions, which limits their application. To address this, encapsulation has become one of the most widely adopted techniques in food processing, transferring the liquid extracts into stable, higher-quality products and improving their stability, solubility, emulsification, and controlled release of BACs with various biological properties. Many studies have shown that encapsulation can even enhance these properties by protecting BACs from severe conditions and facilitating their transport to target tissues or cells where they can exhibit a physiological response [13]. Encapsulation technology plays a significant role in food product development contributing to improved texture, flavor, and color while also extending shelf life. Additionally, the reduced volume and weight of encapsulated dry extracts simplify storage and handling. As the demand for functional foods, dietary supplements, and nutraceuticals continues to rise, encapsulated BACs are becoming increasingly valuable in the industry, driving innovation in health-focused food products.

## 2. Biologically Active Compounds in Berry and Berry By-Products

Berry fruits are widely consumed due to their low calorie content and high nutritional value and are often referred to as “superfoods” or functional foods due to their numerous health benefits. They contain minimal amounts of protein and fat but are rich in dietary fibers (cellulose, hemicellulose, pectin), minerals in traces (manganese), vitamins (ascorbic acid and folic acid), organic acids (citric, malic, tartaric, oxalic and fumaric acids), and various BACs such as phenolic compounds, carotenoids, chlorophylls and phytosterols [5]. Despite their nutritional benefits, berries experience losses at multiple stages of the food supply chain, including harvest, transport, storage, and retail [14]. A significant proportion of berries are processed into fruit juice, generating substantial amounts of waste and by-products, which are highly valuable due to their rich BACs content [15]. Over the years, fruit waste management strategies have evolved, recognizing BBP as an excellent raw material for the extraction of BACs with positive biological effects and the development of high-value-added products. Among BACs, phenolic compounds are the most abundant in berries and berry pomace. However, their distribution within the fruit is uneven: approximately 10% is found in the pulp, 28–35% in the skin and 60–70% in the seeds [16]. The predominant phenolic compounds include flavonoids (flavonols, flavanols, anthocyanins), condensed (proanthocyanidins) and hydrolysable tannins (gallotannins and ellagitannins), and phenolic acids (hydroxybenzoic acid and hydroxycinnamic acid and their derivatives) [17]. Different berry species have distinct phenolic profiles. Blackberries and raspberries of the Rubus genus contain a diverse range of phenolic compounds, while other berry species such blackcurrants (*Ribes nigrum* L.), blueberries (*Vaccinium myrtillus* L.) and chokeberries (*Aronia melanocarpa* L.) are mainly characterized by high concentrations of anthocyanins and proanthocyanidins [5,18]. Anthocyanins are flavonoid pigments responsible for the red, purple and blue color of various berries, and proanthocyanidins as astringent compounds have an effect on taste, mouth feel, and astringency of berries [19]. Six anthocyanidins in berries are differentiated on the basis of various substitutions on the B-ring: delphinidin, cyanidin, pelargonidin, peonidin, petunidin and malvidin; the aglycone basic structure may be glycosylated with sugar units or further acylated with acids. The number of substitutions on the B-ring and the pH value of the cell have an effect on the anthocyanin color of berries [20]. The most important anthocyanins in blueberries are the 3-O-galactoside and 3-O-arabinoside of delphinidin, cyanidin and malvidin, in strawberries is pelargonidin-3-glucoside [21] and in blackcurrants the rutinosides of delphinidin and cyanidin dominate [17]. The anthocyanin concentration of blueberries accounted for 60% of the total phenolic content (TPC) and lowbush species (up to 800 mg/100 g fresh weight (fw)) had higher anthocyanin content than highbush species (more than 1000 mg/100 g fw) [22,23]. Proanthocyanidins occur in berries mainly as monomers of most prevalent flavan-3-ols catechin and epicatechin, but can be present also in various degrees of oligomerization. The concentration of polymers is higher than that of monomers, dimers and trimers, especially in cranberries [24]. Previous studies have shown that the proanthocyanidin content is higher in the early stages of fruit development and decreases as the fruit ripens, while the anthocyanin content increases with ripening [19,20]. Anthocyanins are mainly found in the outer layer of the pericarp [25] and account for about 30% of all phenolic compounds in blackcurrants and about 70% in blueberries, which is why the pomace is a very rich source of these compounds [26]. Chokeberries are considered the richest source of proanthocyanidins, with fresh fruits containing between 522 and 3.671 mg/100 g dry matter (dm), which accounts for 66% to 82% of the TPC of the chokeberry [27]. According to Teleszko and Wojdylo, [28] their content in blackcurrants was 542 mg/100 g dm, while it is about three-fold lower in blueberries (160 mg/100 g dm) [29]. Phenolic acids (hydroxycinnamic acid (HCA) and hydroxybenzoic acid (HBA) derivatives are usually bound to the cell wall or other molecules and are called hydrolyzable phenolic compounds [30]. Blueberries contain significant contents of gallic acid, chlorogenic acid, ellagic acid and ferulic acid; blackcurrants contain p-coumaric acid and chlorogenic acid [31]; and chokeberries are a rich source of caffeic acid, chlorogenic acid and neochlorogenic acid [25]. Compared to blueberries, phenolic acids such as *p*-coumaric acid, caffeic acid and ferulic acid are more abundant in cranberries [20]. In the berries of the genus Rubus (red raspberry, blackberry and cloudberry) and in strawberries, significant contents of ellagic acid have been found (51–88% of the TPC) [32]. Ellagic acid can be considered a dimeric derivative of gallic acid. In addition to their free form, ellagic acid and gallic acid can also be polymerized by esterification with polyols (e.g., glucose) to form ellagitannins and gallotannins (hydrolysable tannins) responsible for astringent sensations and a bitter taste of berry fruits [27]. Berries are also a rich source of mono-, di- and trisaccharide flavonol conjugates, consisting of myricetin, quercetin and kaempferol. Flavonols are substituted as O-glycosides mainly at position 3 of the C-ring by the sugars such as rhamnose, rutinose, galactose, glucose, robinose, rabinose, xylose and glucuronic acid [21]. Blueberries contain significant contents of quercetin (59.4%) and the most significant conjugate sugars were galactoside (35.8–72.1%) and glucoside (12.1–27.1%) [33]. In blackcurrants, myricetin was the main flavonol, followed by quercetin and kaempferol [21]. It is considered that the berry leaves, which are a by-product of the harvest, have a higher phenol content than the fruit and pomace [34,35]. It has also been found that young leaves from the upper parts of the shoots or stems have a higher TPC than their older counterparts from the lower parts [36]. The TPC in different raspberry leaves ranged from 0.88 to 26.196 mg/100 g [34,37] and the flavonoid content in raspberry and blackberry leaves ranged from 0.46 to 1.05% and from 0.14 to 0.31%, respectively [38]. Berry leaves are a valuable source of flavonols, proanthocyanidins and phenolic acids, especially HCA [17]. The most abundant compounds in the leaves are chlorogenic acid and its derivatives [39,40], with concentrations between 59% and 74% of total HCA; [41] found that the concentration of HCA in the bilberry leaves was higher than in the fruit. A total of 33 different compounds were identified in leaf extracts of wild blackberry (*Rubus fruticosus*), including 15 flavonols, 13 hydroxycinnamic acid derivatives, 3 ellagic acid derivatives and 2 flavones [42]. Among the flavonol glycosides, derivatives of quercetin and kaempferol have been identified, with quercetin-3-O-malonylglucoside being the most abundant, although its concentration varies greatly depending on the time of harvest [43]. Blackberry and raspberry leaves have a similar phenolic composition as they belong to the same plant family [44] and have long been used in traditional medicine [34]. In addition to harvest time and genetics, the phenolic content of berries is influenced by many other factors such as environmental conditions (biotic and abiotic), processing, storage and extraction conditions, all of which affect the extraction yield and the variability of phenolic compounds and, consequently, their biological properties [20].

## 3. Extraction of Bioactive Compounds from Berry By-Products

Extraction is a crucial process for isolating BACs from berries and BBP, playing a significant role in the food, pharmaceutical and cosmetics industries. In recent years, extensive research has been carried out, and various new strategies have been developed using advanced extraction techniques that offer potential alternatives to conventional methods such as maceration, infusion, digestion, decoction, percolation, Soxhlet extraction, and shaking. Conventional methods are usually time-consuming and require high temperatures, leading to the degradation of target compounds, the consumption of large amounts of samples and solvents, the production of dilute extracts, and negative effects on the environment and human health [45]. The increasing demand for plant extracts that are safer, more efficient to produce, and cost-effective has driven the development of advanced extraction techniques such as ultrasound-assisted extraction (UAE), microwave-assisted extraction (MAE), pressurized liquid extraction (PLE), high-pressure extraction (HPE), and supercritical fluid extraction (SFE) [46]. These advanced methods offer several advantages, including shorter extraction times, reduced energy consumption, lower environmental footprint, and improved safety, all of which contribute to a more sustainable extraction value chain [47]. The efficiency of extraction depends on several factors, including the extraction technique applied and parameters such as the type and composition of the solvent, the solvent-to-sample ratio, extraction time, temperature, pressure, and power. The selection of an appropriate extraction technique is determined by factors such as the required purity of the extract, the physical and chemical properties of the target compound, its location within the plant matrix (i.e., free or bound), cost-effectiveness and the overall value of the extracted product [48]. To optimize extraction efficiency, conditions must be carefully adjusted to maximize compound recovery while minimizing degradation. Due to the complexity and diversity of polyphenols and their biological matrices, the standardization of extraction protocols remains challenging. Additionally, pre-treatment processes such as drying, homogenization, filtration, and grinding play a crucial role in extraction performance. Conventional extraction methods have been widely applied to the leaves and pomace of various berry fruits for comparison with advanced techniques, generally resulting in lower yields. For example, a lower yield of phenolic compounds was obtained from blueberry, raspberry, blackcurrant, and chokeberry pomace by conventional extraction compared to UAE and/or MAE [49,50,51,52,53], and a lower yield of anthocyanins from strawberry pomace was found with conventional 24 h maceration compared to PLE [54]. An attractive and environmentally friendly alternative to conventional extraction (CE) is UAE, as it is a simple, efficient and cost-effective method. UAE provides improved extraction yield and extracts with higher purity. The interaction of high-frequency sound waves (20–100 kHz) with the plant matrix enables greater penetration of solvents into the plant material due to the cavitation effect on the cell walls and the absorption of sound wave energy, which increases mass transfer. Rupturing the cell walls leads to direct contact with the cell contents, which increases the solubility, diffusion and transport of compounds and improves efficiency [55,56]. Many studies have reported that UAE ensures faster and better extraction of polyphenols with less degradation of the compounds compared to other extraction techniques [57]. The most commonly used ultrasonic reactors in the food industry are ultrasonic baths and/or ultrasonic probes of various lengths, diameters, and shapes, depending on their use [58]. To achieve maximum yield of BACs, process parameters such as probe surface area, electrical amplitude, treatment time, temperature, solvent composition, solvent-to-sample ratio, or the number of extraction steps need to be optimized [59]. Numerous studies have reported the use of ultrasonic baths for phenolic extraction from blueberry [47,49,60,61] and raspberry pomace [60], as well as from berry leaves, such as blackberry [35,62,63], raspberry [37,63,64,65,66,67,68], blueberry [69], and blackcurrant [70]. Ultrasonic probes were used to extract phenolic compounds from blueberry pomace [49,65], blackcurrant [51,53] and chokeberry [51,52,53] and extraction with ultrasonic probes proved to be more effective than conventional methods in terms of shorter time and higher yield of phenolic compounds from blueberry pomace as well as from blackcurrant and chokeberry. The use of an ultrasonic bath also resulted in higher concentrations of phenolic compounds (1.6-fold) from blueberry and raspberry pomace compared to CE [60]. Literature data indicate that changes in extraction temperature and ultrasonic time have a significant effect on polyphenol extraction. For the ultrasonic baths, the temperatures ranged from 20 to 80 °C and the ultrasound times from 5 to 90 min, and for the ultrasonic probes from 50 to 70 °C and from 2 to 35 min. The optimization of temperature (20, 40, and 80 °C) and time (5, 10, and 15 min) for the extraction of phenolic compounds from blueberry pomace in an ultrasonic bath was conducted in the study by Lončarić et al. [61], where the highest concentration of phenolic compounds (5.46 mg GAE/g dw) was achieved at the highest temperature (80 °C) and the longest ultrasonic time (15 min). Optimal parameters for polyphenol extraction from blueberry pomace using an ultrasonic probe were 61.03 °C, 23.67 min, and a solvent-to-sample ratio of 21.70 mL/g in the study by He et al. [49], and 40 °C, 40 min, and 400 W in the study of Zhang et al. [65]. In the study by Elez-Garofulić et al. [52], the optimal UAE conditions for anthocyanin extraction from chokeberry pomace were 25% amplitude and 10 min ultrasonic time, while in the study by Piasecka et al. [53] for blackcurrant and chokeberry pomace, the optimal conditions were 80% amplitude and 10 min. Table 1 presents the reported parameters of UAE of BACs from berry fruit by-products.

In MAE, the energy of microwave radiation (0.3–300 GHz) is used to heat the solute-solvent mixture. The continuous effect on molecules by dipole rotation and ionic conduction leads to an increase in thermal energy and thus extraction efficiency [46]. The advantages of MAE are the reduction in the amount of solvent and extraction time, making the process economically and environmentally sustainable [91]. Polar solvents such as ethanol, methanol and water, which have a high dielectric constant and good absorption of microwave energy, are used for MAE [92]. However, if the temperature of the solution increases too high or too fast during MAE, BACs may be degraded, especially polymeric polyphenols with multiple hydroxyl-like substituents and heat-sensitive polyphenols such as anthocyanins and tannins. For this reason, extraction temperatures and times are set between 50 and 100 °C and from a few minutes to 30 min [93], and MAE is generally used for the extraction of short-chain polyphenols such as phenolic acids and flavonoids [57]. The application of MAE for extracting phenolic compounds from berries has been reported for chokeberry [52], blueberry [65,73], and raspberry pomace [74] as well as for strawberry [75], blueberry [76,77,78,94,95], and mulberry leaves [79]. Considering the importance of various extraction parameters, optimization of the MAE process is necessary to increase the content of polyphenols in the solvent. Usually, statistical approaches such as the response surface methodology (RSM) can be used to find the best operating conditions that provide the relationship between several independent variables or factors and the dependent variables or responses and consequently reduce the number of experiments required, saving time and resources [96]. For chokeberry pomace, optimal MAE conditions were 60 °C for 4 min at 400 W [52], resulting in a significantly higher concentration of TPC (535.8 mg/100 g) compared to UAE (464.8 mg/100 g) and CE (403.4 mg/100 g). A similar observation was made for mulberry leaves, where MAE at 60 °C and 600 W for 20 min resulted in a higher amount of extracted phenolic compounds than UAE (ultrasonic bath, 66 °C, 250 W, 35 min) and CE with reflux (66 °C, 35 min) [79]. Lin et al. [75] determined that the optimal conditions for extracting phenolic compounds from strawberry leaves were: ethanol concentration of 51.1%, extraction time of 40 sec, power of 300 W, and solvent-to-sample ratio of 61.6 mL/g. Furthermore, a 14 min MAE resulted in a higher content of extracted TPC from blueberry leaves (127.06 mg GAE/g dw) compared to a 1 h UAE (97.77 mg GAE/g dw) and 24 h CE (89.16 mg GAE/g dw) [77]. In addition to its efficiency compared to other methods, MAE also shows good selectivity and a significant increase in the yield and purity of the crude extracts [97]. Table 1 presents reported parameters of MAE of BACs from berry fruit by-products.

PLE, also known as accelerated solvent extraction (ASE), is an automated extraction method that combines high temperatures (25–125 °C) and pressures (6.9–13.8 MPa) to keep the solvent in its liquid state during the extraction process. By reducing the surface tension and viscosity of the solvent, the rate of mass transfer is improved and diffusivity is increased [98]. The extraction process is fast (5–25 min) and requires small amounts of solvent (15–45 mL) [99]. Compared to other techniques, PLE is a milder method and is suitable for the rapid and efficient extraction of thermolabile compounds and those sensitive to elevated oxygen concentrations, avoiding the formation of artefacts. Another advantage of this method is the ability to conduct extractions in multiple cycles and to use different solvents or solvent mixtures, which significantly increases the extraction efficiency of the target compounds [99]. In addition, the amount of target compounds is increased when temperature, pressure and extraction time are optimized. For the PLE of phenolic compounds from blueberry [82,86], strawberry [54], chokeberry [83] and cranberry pomace [84], temperatures from 40 to 160 °C, pressures from 50 to 200 bar and extraction times from 5 to 45 min were used. Saldaña et al. [84] optimized the PLE of anthocyanins from cranberry pomace by varying the temperature (40 to 160 °C), solvent type (100% ethanol, 70% ethanol, 30% ethanol, water, water acidified with 5% citric acid) and pressure (50 or 200 bar). Increasing the temperature from 140 to 160 °C led to a decrease in anthocyanin concentration due to thermal degradation of anthocyanins, while no significant difference was observed at lower temperatures. Regardless of pressure and temperature applied, the 100% ethanol proved to be the best solvent for extraction. PLE was more effective than MAE in isolating polyphenols from blackcurrant and bilberry leaves, and an increase in temperature was a critical factor in isolating polyphenols from blackcurrant leaves [81]. In study Terpinc et al. [85], when comparing PLE and MAE, the highest total phenolic content (8027 mg GA/100 g dw) was obtained in wild strawberry (*Fragaria vesca* L.) leaves with optimized PLE conditions (temperature of 150 °C, a static time of 5 min, solvent 30% ethanol solution (*v*/*v*) and a solvent-to-sample ratio of 40:1), and the PLE extract was richer in proanthocyanidins, flavonols, flavan-3-ols, and flavones. The optimal PLE and MAE conditions for polyphenol extraction of black chokeberry leaves were 150 °C, 5 min extraction time and SSR 1:30 g/mL (TPC 80.0 mg GAE/g dm) and 70 °C, extraction time 5 min and SSR 1:30 g/mL (TPC 36.4 mg GAE/g dm), respectively. Both methods yielded similar polyphenol profiles, but MAE extracts contained more flavonols and phenolic acids, while PLE extracts contained more procyanidins and flavan-3-ols Repajić et al. [80]. The study by Pukalskiene et al. [54] showed that regardless of the solvent used, PLE (ethanol at 90 °C and water at 110 °C, 3 cycles of 15 min, pressure of 10.3 MPa) resulted in a significantly higher concentration of anthocyanins from strawberry pomace and at shorter extraction time than conventional maceration (24 h at room temperature on a shaker). Table 1 presents reported parameters of PLE of BACs from berry fruit by-products.

HPE involves the application of pressure in the range of 100–1000 MPa at low temperatures (usually up to 60 °C) with minimal heat-induced degradation of BACs, and is therefore a good alternative to conventional heat treatment. This method is an efficient technology, as it reduces extraction time and solvent consumption and increases extraction yield [45]. The high pressure causes rapid compression of the raw material, which alters its surface and physical properties, leading to rupture of cell membranes and accelerating the mass transfer rate and release of intracellular compounds. Many parameters such as pressure, extraction time, solvent selection and concentration, and solvent to sample ratio can influence the HPE. For example, BACs were extracted from discarded blueberries by HPE (at 500 MPa for 5, 10 and 15 min) and CE with agitation, and the highest content of anthocyanins (117.08 mg 100 g^−1^ fw), polyphenols (9.56 mg 100 g^−1^ fw), and flavonoids (0.62 mg 100 g^−1^ fw) was obtained with HPE for 15 min [87]. HPE performed for 3 min at 500 MPa and room temperature resulted in a higher yield of TPC (70.26%) compared to MAE at 150 sec and 360 W (55.07%) and UAE performed for 1 h at 28 KHz (57.35%) [88]. The optimal parameters for anthocyanin extraction from blueberry pomace were a pressure of 442.96 MPa, ethanol concentration of 63.34%, and a solvent-to-sample ratio of 40.89 mL/g, with fixed extraction time and cycles [88]. Table 1 presents reported parameters of HPE of BACs from berry fruit by-products.

SFE is the most efficient method for the selective extraction of target BACs in the sample fraction, reducing the need for subsequent purification steps [100]. By changing the temperature and pressure, the density of the supercritical fluids can be easily altered so that the selectivity of the extraction can be achieved by applying proper process parameters of SFE. The stability of volatile and thermolabile BACs is increased by the absence of light and air during SFE, resulting in a higher concentration of BACs in the extracts. However, SFE has some disadvantages compared to other extraction methods, such as operation under very high-pressure, high-energy consumption for gas compression, complex solvent regeneration leading to significant energy costs, and high investment costs for equipment [101]. Supercritical extraction usually uses the non-polar solvent carbon dioxide (CO_2_) under high pressure because it is chemically inactive, non-toxic, easily accessible, economical, separable from the extracts, and an approved food-grade solvent [102]. It has both gaseous and liquid properties, a low critical temperature and pressure (31.3 °C at a critical pressure of 72.9 bar), selectivity and the potential to extract heat-sensitive compounds. Low-polarity and small compounds are easily dissolved in supercritical CO_2_ (SC-CO_2_), but large and polar compounds are extracted by adding a co-solvent (ethanol, methanol or water) to increase the extraction yield [103]. SC-CO_2_ has been applied to blueberry pomace [82], chokeberry [83], and strawberry tree fruit [89], using CO_2_ flow rates from 6.3 to 15 g/min, co-solvents such as ethanol (0–20%), water or acidified water, pressures from 5 to 40 MPa, temperatures from 30 to 80 °C and extraction times from 30 to 149 min. Under optimal SFE conditions (60 bar, 48 °C and ethanol concentration of 19.7%), a higher TPC of strawberry tree fruit (25.72 mg GAE/g) was obtained compared to conventional water extraction (24.89 mg GAE/g) and ethanol extraction (15.12 mg GAE/g) [89]. The highest yield of phenolic compounds and anthocyanins from chokeberry pomace was achieved under optimal conditions of SC-CO_2_ extraction at 35 °C, 10 MPa, and 80% ethanol addition [90]. In the study by Paes et al. [82], a higher concentration of TPC (134 mg GAE/g) was extracted from blueberry pomace with SC-CO_2_ extraction (20 MPa and 5% ethanol and water as co-solvents) than with PLE with 50% ethanol (90 mg GAE/g). Table 1 presents reported parameters of SFE of BACs from berry by-products.

When extracting raw materials on a large scale, economic efficiency and productivity must be fully taken into account, and the scaling of extraction processes is the subject of numerous research projects. About 60% of industrial applications are carried out with SFE, 15% with ultrasound and 14% with microwaves [48]. UAE and HPE, as non-thermal technologies with mild temperatures and shorter extraction times, are the most commonly used extraction methods for industrial juice production [104]. The combination of UAE with other extraction technologies is focus of research and can bring further benefits [105]. However, in the case of the PLE extraction process, there are still challenges for the industrial scaling of the process [106]. The extraction and utilization of BACs from by-products of the fruit industry alleviates the environmental problem and reduces the burden of waste disposal. Significant differences in the structure and biological activity of BACs affect the choice of extraction method, which ensures the highest yield of the target compound with minimal degradation [107].

## 4. Biological Effects of Berry By-Products Extracts

Polyphenols are the major BACs of berry wastes and BBPs, and have received considerable attention due to their health-promoting properties, including anti-inflammatory, antioxidant, antiviral, antimicrobial, antiallergic, anticarcinogenic, antihypertensive, and immunomodulatory effects, etc. [5,108]. In addition, polyphenols protect against chronic diseases (diabetes, cardiovascular and neurodegenerative diseases) and reduce the risk of certain types of cancer [5,109]. Chemical interactions between phenolic compounds and with other dietary components can have significant effects on their biological activities as they act synergistically or additively [110]. For example, BBPs and berry seed oil contain dietary fiber and polyunsaturated fatty acids [111], which also have bioactive properties, and are therefore considered as functional ingredients due to their effects on human health [112]. Among the bioactivities of phenolic compounds, the antioxidant activities have been widely studied, including the ability to scavenge free radicals, chelate the ions of metals that trigger oxidation processes, inhibit lipid oxidation, reduce the formation of hydroperoxides, etc. [113]. Free radicals play an important role in various physiological conditions, but their excessive production can cause the development of various diseases. Protection against free radicals can be enhanced by the intake of dietary antioxidants, which can be of great importance for disease prevention. A number of in vitro methods with different reaction mechanisms can be used to determine the antioxidant capacity, such as hydrogen atom transfer (ORAC), single electron transfer (FRAP) or their combination (DPPH, ABTS). Among the free radical scavenging methods, the DPPH method, which is fast, simple and inexpensive, and the ABTS method, which is suitable for both hydrophilic and lipophilic antioxidants, are the most commonly used [114]. The antioxidant capacity values of pomace of selected berries determined by the DPPH and ABTS methods are shown in Table 2.

The pomace contains a considerable phenolic content, about 28–35% in the skin, 60–70% in the seeds and 10% in the pulp [121], and the antioxidant capacity of berry pomace can be higher than that of fresh berries. For example, a higher antioxidant capacity was found for chokeberry pomace (DPPH 3.01 µmol Trolox equivalents (TE)/g dm; ABTS 7.79 µmol TE/g dm) compared to fresh berries (DPPH 2.79 µmol TE/g dm; ABTS 4.39 µmol TE/g dm) [115]. Similar results were also confirmed for chokeberry pomace [116,117] and blueberry pomace [118] compared to fresh fruits. The literature indicates that the pomace has a different antioxidant capacity depending on the variety of red berries, and anthocyanins are probably the main polyphenol class responsible for the observed antioxidant activities, as the skin and seeds have a significantly higher content of these compounds than the pulp [122]. The chemical structure of anthocyanins determines their capacity and effectiveness as antioxidants [122]. Compared to other polyphenols, anthocyanins remain mainly in the pomace, as they are associated with the antioxidant fractions of the cell wall, which are less soluble in water, and are surrounded by a hard shell in the seeds of the berries, and their extraction requires further processing. In addition to their ability to scavenge free radicals, due to their high chemical diversity, berry polyphenols also have antimicrobial effects [123,124,125,126]. Strong antibacterial properties are found in berries with a high tannin concentration, such as chokeberries, blueberries, raspberries, and strawberries. The antibacterial mechanism of the polyphenolic compounds depends on the permeability of the outer and plasma membrane of the pathogen to allow hydrophobic compounds to enter the bacterial cell by destroying the lipopolysaccharide layer. Due to differences in the cell wall structure, Gram-negative bacteria are less sensitive to flavonoids than Gram-positive, whose phospholipid bilayer structure is more easily damaged [127]. Additional polyphenolic mechanisms include the effects on microbial metabolism, the inhibition of extracellular microbial enzymes and the elimination of substrates required for microbial growth [128]. According to a study by Meremae et al. [4], ethanol extracts of chokeberry and blackcurrant berries and their pomace had the highest content of quercetin derivatives and anthocyanins, showed a high antioxidant capacity and inhibited the growth of Gram-positive bacteria such as *Staphylococcus aureus* and *Listeria monocytogenes*. The suppression of the growth of microorganisms is also influenced by the low pH values of the pomace [129], and the addition of powdered berry pomace for example, to meat products ensures high microbiological quality [4]. The phenolic compounds present in foods reduce also lipid oxidation [130], which can be caused by free oxygen radicals or reactive oxygen species and thus change the texture, taste or odor of foods and shorten their shelf life. The influence of blueberry pomace as a natural ingredient on the oxidative stability of pork patties during cold storage and baking was investigated in a study by Peiretti et al. [131]. The results showed desirable changes in the cooking properties of the patties and reduced concentration of volatile oxidation compounds. In addition, the phenolic compounds of the berry pomace also have a potential prebiotic effect, as previous studies have shown that the phenolic compounds of berries have a positive effect on intestinal health and promote the growth of beneficial microorganisms [132,133]. Berry leaves can also be considered as an alternative source of BACs with health-promoting properties, especially since the use of berry leaves in traditional medicine has been known since ancient times. Numerous studies confirm various biological effects and a possible application for the prevention and alleviation of symptoms of various diseases [5]. For example, black currant leaf tea is a traditional remedy for relieving joint pain and is used as a diuretic for mild urinary problems, against diarrhea and spasmodic coughs, and for diaphoretic properties [17,134]. Since ancient times, blackberry leaves have been thought to have beneficial effects on health and strengthening the immune system, and Hippocrates recommended blackberry stems and leaves soaked in white wine to facilitate childbirth [135]. Zia-UI-Haq et al. [136] state that a decoction of blackberry leaves was traditionally used as a tonic and mouthwash in the treatment of oral candidiasis, gingivitis, sore throat and mouth ulcers. According to Oszmiański et al. [40], blackberry leaves and roots are traditionally used for respiratory ailments, but also used as a traditional remedy for anemia and gynecological disorders, diarrhea, dysentery, cystitis and hemorrhoids. Blackcurrant leaf also has significant antioxidant and anti-inflammatory properties (inhibition of myeloperoxidase activity and the production of reactive oxygen species on activated neutrophils) [137,138]. The potential anti-inflammatory effect of the BACs of the dry extract of blackcurrant leaves has been also documented in the study of Garofulić et al. [81]. Certain condensed tannins and prodelphinidin from blackcurrant leaves have been shown to inhibit the inflammatory enzymes COX-1 and COX-2, and are therefore effective in the prevention of osteoarthritis and have a positive effect on joint health [139]. In addition, the potential analgesic properties of blackcurrant leaves were demonstrated in studies on carrageenan-induced paw oedema in rats, which was later confirmed by the inhibition of leukocyte infiltration by blackcurrant proanthocyanidin [134]. Blackberry leaf extracts showed antidiabetic antioxidant and angiogenic effects [17,36,42,140,141,142] as well as significant antimicrobial activity against several bacterial strains such as *Salmonella typhi*, *Escherichia coli*, *Staphylococcus aureus*, *Micrococcus luteus*, *Proteus mirabilis*, *Bacillus subtilis*, Citrobacteria sp. and *Pseudomonas aeruginosa*, which is greater than that of fruit [136]. The extracts from raspberry leaves showed a significant and stronger antioxidant capacity than the extracts from blackberry leaves [36]. The medicinal properties of raspberry leaves are attributed to the high content of hydrolyzed tannins, ranging from 2.6 to 6.9%, the most abundant being derivatives of ellagic acid [142]. Raspberry leaves are a traditional herbal remedy for the symptomatic relief of mild cramps associated with menstruation and gynecological complaints and diseases, for the symptomatic treatment of mild inflammation of the mouth and throat and for the treatment of gastrointestinal complaints diarrhea [138,143,144]. It has been reported that hot tea infusion of raspberry leaf induces labor [138,145] and is traditionally used as an astringent and, less commonly, for chronic skin conditions and to treat conjunctivitis [17]. The aqueous extracts of bilberry leaves showed antidiabetic, anti-inflammatory (oral cavity) and antibacterial effects [36]. For example, ref. [146] investigated the use of a dried hydroalcoholic extract of bilberry leaves in streptozotocin-diabetic rats (3.0 g extract per kg body weight) and found a biologically significant 26% reduction in plasma glucose levels and a lipid-lowering effect, i.e., a 39% reduction in blood triglycerides in animals with dyslipidemia. Ref. [147] tested a multicomponent herbal antidiabetic agent patented in Croatia, consisting of ten plant species, including bilberry leaves, and the results showed a reduction in blood glucose and fructosamine levels in alloxan-induced non-obese diabetic mice. Decoctions and infusions of bilberry leaves are also used for their diuretic, astringent and antiseptic effects on the urinary tract. Sadowska et al. [148] also investigated the antistaphylococcal activity of bilberry leaves, which simultaneously increased the bactericidal potential of vancomycin and linezolid in combination. The anticancer potential of bilberry leaves was also investigated, with flavonoids as well as caffeic acid and chlorogenic acid extracted from the leaves of the Sakhalin bilberry *Vaccinium smallii* and studied as anticancer agents. The results showed inhibition of neoplastic transformation of mouse cells induced by epidermal growth factor without toxic effects [149]. Recent studies have shown that extracts from berry leaves have an antileukemic effect against sensitive HL60 cells in vitro [150], and the aqueous extract of strawberry leaves acts as a direct, endothelium-dependent vasodilator whose effect is mediated by nitric oxide and cyclooxygenase products [151]. Phenolic extracts from the leaves of black chokeberry protected against oxidative and DNA damage, but also induced free radical formation and DNA damage at different doses. The extracts also had moderate antimicrobial activity against S. aureus and B. subtilis and no antimicrobial activity against Gram-negative bacteria as well as yeasts, lactic acid bacteria and molds [80].

A summary of the traditional medicinal uses and significant biological effects of selected berry leaves demonstrated in vitro, in vivo and in clinical studies is presented in Table 3. In view of the abovementioned studies, the leaves and pomace of the berries can be considered a source of valuable BACs with health-promoting and disease-preventing properties.

## 5. Encapsulation in the Processing of Berry By-Products

The efficacy of BACs depends on maintaining their stability against external factors such as heat, light, oxygen and moisture during processing and storage, as well as ensuring their bioavailability and bioactivity [164,165]. BACs derived from natural sources usually have an unpleasant taste that limits their use, but also exhibit a low bioavailability, as they are susceptible to degradation by the low pH in the gastric environment. To effectively compensate for these deficiencies, numerous encapsulation methods have been developed to ensure the protection of BACs from harmful environmental influences, enable their prolonged stability during processing and storage, facilitate their incorporation into food matrices, mask possible undesirable tastes and odors, facilitate their transport to target tissues and increase their bioavailability through controlled release, thereby enhancing their bioactivity. In encapsulation, the functionally active ingredient, called the internal phase, core or filling in the form of solid, liquid or gaseous particles, is enclosed in a membrane called a shell, capsule or carrier [166,167]. The choice of carrier and encapsulation technique depends on the properties of the active ingredient and the desired application, as the stability of the active ingredient largely depends on the structure and functionality of the carrier, the processing conditions and possible interactions between the active ingredient and the carrier [168]. Common encapsulation materials include carbohydrate polymers, proteins and lipids with GRAS (Generally Recognized As Safe) status that can be used alone or in mixture. Encapsulation materials based on carbohydrates are starch and starch derivatives (maltodextrins, cyclodextrins), cellulose and cellulose derivatives (ethylcellulose), plant extrudates and extracts (gum arabic, pectins, galactomannans), extracts from seaweed (alginate and carrageenan) and carbohydrates of microbial and animal origin (dextran, chitosan, xanthan and gellan). Encapsulation materials based on proteins of plant origin are gluten and soy isolates as well as whey protein isolates of animal origin, gelatine and casein, while encapsulation materials based on lipids are phospholipids and waxes (beeswax, carnauba wax) [169]. Among these, maltodextrin is the most commonly used carrier material, followed by gum arabic, due to their suitability for different drying processes and wide application potential [170]. Depending on the properties and the type of contact between the active ingredient and the carrier, different encapsulation techniques have been developed, which can be divided into three basic groups: mechanical (spray drying, spray cooling, freeze-drying, fluidized bed drying, extrusion techniques, encapsulation with supercritical fluids), physico-chemical (ionic gelation, coacervation, encapsulation in liposomes) and chemical (polymerization, molecular entrapment) [171]. The choice of encapsulation technique depends primarily on the properties of the BACs, the type of carrier used and the final use of the product, but in the food industry, spray-drying and freeze-drying are the two most commonly used techniques for encapsulating BACs to produce functional food ingredients, ensure controlled release and utilize the biological potential of food waste [172]. Freeze-drying is a low-temperature and low-pressure dehydration process that removes the water in the form of vapor, but the process is relatively slow and expensive, while spray-drying allows rapid evaporation of the water and maintains the low temperature in the particles [173]. Most of the research relates to the encapsulation of BACs from pomace, as the majority of waste from berry processing consists of this form of by-product. Table 4 provides an overview of research on the encapsulation of BACs from berry waste from pomace and leaves. The most common method for encapsulating BACs from berry pomace is spray drying. For example, when blueberry pomace extract was encapsulated by spray-drying, 98% and 70% of anthocyanins were retained when maltodextrin [174] and whey protein isolate were used as carriers [175], respectively. While whey protein isolate resulted in a low moisture content (≈5%), it was less effective in protecting anthocyanins from degradation during storage compared to other carbohydrate carriers [175]. From an industrial standpoint, spray drying has certain advantages over other techniques. For example, ionic gelation and co-crystallization [176,177] have also been used for the encapsulation of BACs from berry pomace, but compared to spray-drying, the efficiency of these methods was lower in terms of retention of TPC and the capsules had a significantly higher moisture content. Apart from the increased hygroscopicity, the powders produced by co-crystallization (−24.75%) and ionic gelation (−52.97%) showed the lowest stability of the phenolic compounds during one-month storage compared to spray drying (−6.07%) [177]. The combined application of emulsification (in the form of a double water-oil-water emulsion) and freeze-drying for the encapsulation of phenolic compounds from chokeberry pomace extract carried out by Eisinaitè et al. [178] resulted in powders with low moisture content (1.87%), attractive color and good reconstitution properties. This combined process showed a high encapsulation efficiency (95.36%), and in the reconstituted form the powders showed a high potential for use in the confectionery industry as a value-added ingredient due to the creamy structure of the emulsion. In addition to the berry pomace, the leaves of the berry species also showed important biological effects due to the high concentration of BACs, but their great potential as a raw material is not sufficiently utilized. For example, extracts of bilberry leaves encapsulated by nanoprecipitation showed promising results in the treatment of depression, as their efficacy in reducing depressive symptoms was comparable to that of antidepressants [179]. Encapsulated pomace extracts are most commonly used in functional foods or as natural colorants, while encapsulated extracts from berry leaves are more commonly used as dietary supplements due to their therapeutic effects. Encapsulation of phenol-rich leaf extracts of various species of the genus Vaccinium and Morus by spray- and freeze-drying significantly increased their bioavailability compared to liquid forms and enabled their use as functional food supplements but freeze-drying results in better retention of the phenolic compounds [180,181,182]. An innovative strategy to improve the stability, delivery and bioavailability of most BACs and for controlled release at specific sites is the use of liposome systems [73]. In the study by Păvăloiu et al. [183], the encapsulation of polyphenols from the leaves of the goji plant in liposomes was performed and the results showed an encapsulation efficiency of up to 85%, good storage stability over 3 months at 4 °C and a cytoprotective effect on fibroblast cells. Therefore, liposome encapsulation could be considered as a great strategy for the delivery of polyphenols. The results so far indicate a great potential for the application of encapsulated extracts from berry waste in the food and pharmaceutical industries. However, as numerous factors contribute to the utilization of berry waste, such as the application of encapsulation techniques, the selection of the optimal technique depending on the raw material and the type of active molecules and final products, as well as the industrial use of these results, further research is needed.

## 6. High Value-Added Products from Berry By-Products

The use of BBPs is gaining increasing interest in the food industry as it adds value to food by improving antioxidant capacity, microbiological and oxidative stability and nutritional profile, and contributing to a more sustainable food chain and the circular economy. This chapter discusses the use of BBPs for food fortification and preservation. Berry pomace can be incorporated into food products in various forms, including fresh or dried ingredients, liquid or dry extracts, or powders obtained through encapsulation techniques. Dried and powdered pomace is commonly added to smoothies, bakery products, confectionery, functional products, and teas [184]. For example, incorporating fresh and dried blackcurrant or redcurrant pomace into apple juice-based smoothies significantly increased their phenolic content, antioxidant and antidiabetic in vitro activity compared to the original product, while maintaining positive sensory properties [185]. The use of by-products for the production of infusions also seems to be an interesting type of utilization, not only as an addition to teas, but also as their basis. Studies have shown that the infusion made from dried chokeberry pomace contains more polyphenols and has a better flavor and color than the infusion made from the whole fruit [186]. The consumption of baked bakery products is traditionally high, but these products have a high energy density and contain a lot of carbohydrates and fats. The addition of berry pomace to baked and extruded bakery products or snacks enhances their nutritional profile and increases the content of phenolic compounds, vitamins, minerals and dietary fiber [187]. For example, incorporating chokeberry pomace in shortcrust pastry sweetened with sucrose and erythritol improved its nutritional, health-promoting and sensory properties, which can be accepted by potential consumers with carbohydrate metabolism disorders and health-conscious consumers [184]. Using BBPs to develop baked goods can also offer economic benefits to the food industry by eliminating waste disposal costs and transforming BBPs into value-added products. It was found that the antioxidant capacity of cookies with butter and freeze-dried snack products was significantly higher when berry pomace was added, as well as the protein and ash content, and the content of potentially bioavailable polyphenols [187]. The partial replacement of wheat flour with berry pomace flour is an effective strategy for reducing the glycemic index of cookies. For example, biscuits made with blackcurrant or chokeberry pomace had lower glycemic index values compared to traditional biscuits [188], and the results of in vitro digestion showed that the addition of blackcurrant pomace to extruded flour muffins had an inhibitory effect on enzymes for digestion of starch, which reduced the release of glucose from pre-gelatinized starch and neutralized the hyperglycemic effect caused by flour extrusion. The enrichment of bakery products with berry pomace without impairing their sensory and physicochemical properties has been the focus of numerous studies. Several studies have shown that the addition of BBPs to baked goods such as muffins and cookies, up to a certain level of substitution, improves the nutritional profile and does not affect the sensory properties of the product. For example, replacing 10% of the typical flour (wheat, rice and corn) with chokeberry pomace flour in the dough results in products that are sensory acceptable to potential consumers, while a higher replacement reduces the overall quality [189]. Šarić et al. [190] investigated the addition of blueberry and raspberry pomace in the production of gluten-free cookies, where the highest acceptability was achieved by replacing the gluten-free flour mixture with 28.2% blueberry pomace and 1.8% raspberry pomace. The cookies produced according to the optimized formulation had a similar protein and carbohydrate content, but a significantly lower fat content than cookies with gluten. A daily portion of cookies for adult men and women was found to provide 5% and 7.73% of the recommended daily intake of linoleic acid, 23.6% and 34.3% of α-linolenic acid, and 10.3% and 15.6% of dietary fiber. The addition of chokeberry pomace polyphenol extract to cookies had an effect on reducing lipid oxidation in the production of cookies and thus extended shelf life [191]. When evaluating the nutritional value of bakery products enriched with berry pomace, the influence of heat treatment and the conditions applied during baking on the stability of the BACs present should be taken into account. During thermal processing, anthocyanins are the most unstable and flavonol glycosides the most stable phenolic compounds in berry pomace. The combination of higher temperature and shorter baking time had the best effect on the preservation of polyphenols. However, as the composition of polyphenols in different fruits varies, it is important to optimize the baking conditions for the different types of pomace [192]. From a technological point of view, pomace added to bakery products has the function of a filling due to its high dietary fiber content, as it increases the mass of the product and reduces the sugar, flour or fat content, which enables the production of low-calorie products [187]. On the other hand, the addition of berry pomace can lead to an impairment of certain technological properties of baked products, in particular texture and volume. Berry pomace contains a higher proportion of insoluble fibers (cellulose, hemicellulose and lignin) than soluble fibers, which leads to a reduction in the hydration of wheat proteins and a weakening of gluten strength due to the porosity of insoluble fibers that facilitate water absorption [193]. Due to the above effects, the addition of berry pomace to bakery products can lead to lower aeration, longer development time, smaller volume and firmer structure of the product. According to Struck et al. [194], the partial replacement of wheat flour by the addition of blackcurrant pomace resulted in stiffer, less elastic and stretchy doughs, but with the addition of up to 10% dried blackcurrant pomace, products with satisfactory properties were obtained. To minimize the negative effects described, it is recommended to prehydrate the pomace in hot water for 30 min, which improves the processing properties of the dough and results in a satisfactory quality of the bread produced. The appropriate amount of water for prehydration can be determined by trying to achieve a similar kneading resistance in farinograms [195]. The partial replacement of flour, fat and sugar with blackcurrant or chokeberry pomace had different effects on the preparation of the cookies, depending on which ingredient was replaced. For example, the cookies in which the flour was replaced had a lower height and hardness, a smaller number of pores, but a larger diameter. Cookies in which the fat was replaced had a lower mass loss during baking and a greater height than the cookies in which the flour was replaced, and the cookies in which the sugar was replaced generally had the highest values for the texture parameters and a higher fraction of air pores with a smaller diameter. The most acceptable cookies to consumers were those in which flour and fat were replaced with berry pomace [188]. Schmidt et al. [196] investigated the use of blackcurrant pomace (10%, 20% or 30%) as a partial substitute for flour in the production of crackers and found that it significantly affected the physical properties of the dough (extension, adhesiveness, dynamic rheology in simulated baking) and the properties of the baked product (color, texture and sensory properties). Depending on the degree of substitution, the addition of pomace resulted in a significantly softer and less extensible dough, which can be attributed to a less pronounced protein network due to possible fiber–protein interactions or a lower hydration of the flour components. The weaker protein network of the pomace formulations led to a lower increase in volume during baking and a lower hardness. In contrast, dough handling and sensory acceptability were not affected by the use of pomace, although the color of the final product was greatly altered. Sensory evaluation and the development of new products are closely linked, as changes in sensory properties when adding berry pomace to baked products can influence consumer acceptance of the product [197]. For example, the addition of 20% berry pomace to cookies resulted in a darker color with a higher proportion of yellow color tone, and it was observed that such a color of the product can attract consumers. In the study by Kitchen [198], an extruded snack product of sorghum and blueberry pomace powder was purple and darker in color, had a smaller volume, higher bulk density and hardness compared to a control sample without blueberry pomace powder, but the snacks were uniformly preferred by consumers. In addition, the snack with blueberry pomace powder contained more dietary fiber, anthocyanins, phenolic compounds and higher antioxidant activity. The addition of blackcurrant pomace as a flour substitute in muffins did not result to significant differences in texture or consumer acceptance compared to a standard formulation, but counteracted the hyperglycemic effect of pre-gelatinized starch [199]. Regarding other sensory properties, according to Curutchet et al. [200], the results of the acceptance of cookies enriched with blueberry pomace showed that the influence of taste could outweigh the influence of health benefits. On the other hand, a study by Reißner et al. [201] found that the use of pomace in food products was rated remarkably positively, highlighting consumer awareness of healthy longevity and sustainability, and that the overall acceptability of crackers with added blackcurrant pomace was significantly related to the country of origin and gender of respondents. Yoghurt is one of the most widely consumed fermented milk products in the world due to its nutritional value, but the content of phenolics and other BACs in milk is generally low [202]. Therefore, the addition of BACs from berry pomace offers a good opportunity to increase the nutritional potential of dairy products. The berry pomace is usually added in the form of powder or extract, as strong sedimentation occurs when the pomace is added directly to liquid and semi-liquid products. Studies on the addition of berry pomace powder have shown a positive effect on the physico-chemical and textural properties of yoghurt. For example, the addition of 5% blackcurrant pomace powder to yoghurt leads to an improved texture due to the high proportion of soluble fiber contained in the pomace and consequently to a high water-retention capacity [203]. The addition of up to 3% mulberry pomace significantly increased the water-retention capacity, reduced the firmness of the yoghurt and had a positive effect on the consistency and viscosity of the yoghurt [204]. Dos Santos et al. [50] investigated the bioavailability of anthocyanins from yogurt containing blackberry pomace microcapsules prepared by different drying methods (spray-drying, freeze-drying, ionic gelation) after in vitro digestion. Yoghurt preparations with spray-dried microcapsules showed a higher bioavailability of cyanidin-3-glucoside than microcapsules prepared by freeze-drying or ionic gelation. In addition, the yoghurt formulations showed higher bioavailability after gastrointestinal digestion than the pristine microcapsules. In the study by Raikos et al. [205], blackcurrant pomace extract at a concentration of 20% (*w*/*w*) was used to fortify yoghurt beverages with antidiabetic properties, and an increase in TPC was observed after 4 weeks of storage. Research by Ni et al. [206] showed that addition of blackcurrant pomace extract to yoghurt beverage increases the phenolic content and antioxidant capacity, and can also induce an antidiabetic effect in vitro. The beverage formulated with blackcurrant pomace extract showed a higher potential to inhibit the activity of α-glucosidase compared to salad berry pomace, possibly due to the peptides released from the caseins during the fermentation process. The addition of blueberry pomace improved the overall acceptability and sensory properties of buttermilk enriched with 2% and 4% blueberry pomace [207]. Fermentation of blueberry pomace by the probiotic *Lactobacillus casei* has been shown to increase antioxidant activity and regulate the fecal microbiota, which is potentially beneficial for health [208], and BACs from blueberry pomace exhibit significant antimicrobial activity, which offers potential for innovative natural food additives [209]. Therefore, in addition to dairy products, berry pomace has great potential for use in the production of meat products, as the enrichment with pomace introduces natural compounds with antioxidant and antimicrobial potential and reduces the use of synthetic additives while increasing nutritional value. Babaoglu et al. [210] reported that the water extract of chokeberry pomace could be a promising natural preservative among the pomace extracts of various berries to improve oxidative stability and increase the microbiological quality of beef patties during refrigerated storage. The antimicrobial effect of phenols contained in berry fruits and their pomace against bacterial pathogens in the gut has been extensively studied, and BACs from berry pomace significantly reduce the growth of bacteria by altering their properties and motility. Proposed mechanisms of microbial inhibition by berry phenols include damage to the bacterial cell membrane, inhibition of extracellular microbial enzymes, disruption of microbial metabolism and deprivation of substrates required for microbial cell proliferation and pathogenicity [211]. Tarasevičienė et al. [212] investigated the effects of the addition of raspberry pomace and blackcurrant at concentrations of 1%, 3% and 5% on the freshness of beef burgers during a 9-day storage period at 4 °C. The addition of pomace, even at the lowest concentrations, acted as an effective antioxidant and reduced lipid oxidation, but also increased the fiber content and acted as a thickening agent. In another study, Tamkutė et al. [213] investigated the addition of 2% defatted chokeberry pomace extract to improve the stability of pork products, and the results showed a reduction in oxidation, an extension of the shelf life and no negative effects on the overall sensory quality of the meat products. Other possible applications of berry pomace compounds (anthocyanins and other (poly)phenols) are the production of edible films and active packaging, as well as colorants for the food industry due to their bioactive properties (antioxidant and/or antimicrobial) and the chromatic transitions. The use of berry-bioactive impregnated starch-based films to extend the shelf life and quality of fresh and minimally processed fruits and vegetables, bakery products, dairy products, meat, fish, confectionary and space food has been increasingly researched in recent years [214]. Singh et al. [215] developed edible starch-based packaging films containing blueberry pomace powder and investigated their suitability for food packaging. Edible films with a higher proportion of pomace showed improved light-barrier properties, suggesting a positive effect in preventing UV-induced spoilage of food. This active packaging retained its structural integrity and exhibited high tensile strength and can therefore be used for a variety of food products, especially low to medium water content products, where the release of BACs from blueberry pomace can increase the nutritional value of the packaged product. Chitosan and pectin films enriched with 10% and 20% (*w*/*w*) blackcurrant pomace powder have also been developed to reduce losses during food production and improve the functional properties of these films intended for coating or wrapping [216]. The results showed that the water vapor permeability of the active films increased (by up to 25%) and indicated a higher moisture content retention (27%), especially in pectin-based films, which could be useful for maintaining the freshness of food. The water solubility was not significantly altered, but the mechanical properties (tensile strength, elongation at break and Young’s modulus) were mainly reduced by the residual insoluble particles present in the blackcurrant waste. The antioxidant activity of the blackcurrant pomace-enriched active films was increased up to 30-fold, and they can potentially be used to extend the shelf life of food products. Edible coatings containing blueberry pomace showed higher levels of color intensity [217]. In addition, significant color changes have been observed especially in chitosan film formulations after exposure to different pH buffers, which could be used as color change indicators for intelligent bio-packaging to improve food quality monitoring and reduce food waste [216]. The latest trend is the use of new edible coatings enriched with extracts from berry leaves to increase the antimicrobial and antioxidant activity of the edible coatings and thus improve the quality and extend the shelf life of fresh and minimally processed fruits and vegetables. For example, when blueberry leaf extracts were incorporated into a chitosan coating, the antimicrobial function of the coatings was improved and can be used to extend the shelf life and maintain the high nutritional value of fresh blueberries during post-harvest storage [218]. Other functional ingredients, such as nutraceuticals, flavor, and color agents from BBPs can be carried by edible coatings and retained on the food surface to improve food quality, stability, and safety but possible negative effects are the development of undesirable sensory properties of the coated products. One of the upcoming trends in the industry is also the valorization of berry pomace as a suitable raw material for the production of anthocyanin-rich powders, which are suitable for improving the color and nutritional value of food. According to Nemetz et al. [219], blackcurrant, blueberry and elderberry pomace powders have promising techno-functional properties, but their use in low-viscosity solutions is limited due to their excessively high sedimentation rates. Despite the higher dosage required to achieve comparable colors to other food colorants, the potential of berry pomace powders for industrial use is increasing as it is a cost-effective coloring food with additional benefits in terms of further increasing fiber and phenolic content in food applications. The use of berry pomace powders shows a wide applicability in bakery or dairy products and in products with a longer shelf life, as storage tests suggest their acceptable color stability. Despite the numerous advantages, the use of BBPs in food production is associated with various challenges, such as the increased swelling capacity of fiber-enriched doughs or the increased sedimentation of pomace in liquids and potential sensory alterations. Therefore, future research should focus on optimizing processing techniques tailored to different BPPs and food products to maximize benefits while minimizing drawbacks. With ongoing advancements, the industrial application of BBPs is expected to expand, promoting sustainable food production and reducing waste. Food safety is a crucial concern in the food industry; however, most studies do not provide a comprehensive safety assessment (e.g., microbiological analysis, physicochemical quality evaluation or contaminant determination) of these innovative products derived from BBPs. The use of extracts, flours, or powders from dried BBPs raises safety concerns, including potential contamination with pathogens, pesticides, and heavy metals, as well as the presence of anti-nutritional compounds such as oxalates, tannins, saponins, and alkaloids. Additionally, these products may be prone to rapid auto-oxidation [220]. Effective strategies to control microbial contamination include heat treatment, ionizing irradiation, pasteurization, and sterilizing filtration of BBP-derived extracts. Heat treatment and gamma or ultraviolet irradiation can also reduce anti-nutritional compounds to safe levels, though they may, in some cases, lower BAC content. A comprehensive approach combining good agricultural and manufacturing practices, proper storage conditions, environmental controls, post-harvest biosecurity methods, and quality assurance programs—such as the HACCP system—can enhance the safety and industrial use of value-added BBP ingredients. Additionally, ongoing education for consumers and the food industry, along with prompt regulatory responses from policymakers and legislators, is essential. Strict new regulations should be developed to govern the use of fruit waste as food ingredients, ensuring both safety and sustainability.

## 7. Conclusions

The application of berry by-products as functional flours and sources of BACs and health-promoting antioxidants presents new opportunities for waste management in the fruit industry. However, effectively valorizing these by-products remains a challenge for the global food industry, since the yield and the variability of BACs and consequently their biological properties are influenced by the diversity of fruit species, variability among cultivars, seasonal production fluctuations, extraction conditions, storage and the complexity of processing methods used to develop final products. To enhance the commercial application of berry by-products, further efforts are needed to develop stable liquid and dry extracts with high bioactive content, suitable for industrial applications and possessing antioxidant and antibacterial properties. The raw material, the type of active molecules, final products, and the industrial use have an impact on the selection and optimization of encapsulation techniques. Despite the numerous challenges associated with the properties of the final products (physico-chemical and possible sensory alterations) and safety concerns, the industrial application of BBPs is expected to increase in order to promote sustainable food production and reduce waste. Additionally, in response to environmental concerns, innovative packaging solutions utilizing berry by-products have emerged as a sustainable approach to preserving food quality while maintaining physico-chemical and sensory attributes of food. Future trends should focus on expanding research into biodegradable and intelligent packaging materials made from berry waste-derived biopolymers. It is predicted that the acceptance and consumption of foods enriched with fruit by-products will continue to grow among consumers. However, these products must be nutritious, sensorially appealing and affordably priced. Clear communication regarding their safety and health benefits will be essential in driving consumer trust and adoption. The role of berry by-products in personalized nutrition, gut microbiota modulation and development of functional foods tailored to specific health conditions like diabetes, obesity or cardiovascular diseases presents a future challenge for researchers. Moreover, the nutritional and functional properties of berry pomace could be additionally improved by exploring new microbial and enzymatic fermentation strategies. In summary, further measures need to be taken with regard to implement new technologies for large-scale industrial production of functional foods incorporating berry by-products, to assess safety (through physico-chemical, microbiological and toxicological studies) and to adopt specific regulations to ensure the safety of utilized berry by-products.

## Figures and Tables

**Table 1:** Reported parameters of advanced extraction methods of bioactive compounds from berry fruit by-products.

Plant Species	Solvent	Extraction Parameters	Total Phenolic Content (mg GAE g^−1^ dw)	References
UAE				
*Rubus idaeus* (leaves); *Rubus fruticosus* (leaves)	70% MeOH	room temp.; 1 h	144.20 ± 1.58; 132.90 ± 3.33	[63]
*Vaccinium angustifolium* (pomace)	0, 10, 50, 90% EtOH	20, 40, 60 °C; 30, 60, 90 min	6.31 ± 0.15	[47]
blueberry (pomace)	50% EtOH + 1% HCl; 50% MeOH + 1% HCl	20, 40, 80 °C; 5, 10, 15 min	10.52	[61]
*Vaccinium myrtillus* (pomace); *Rubus idaeus* (pomace)	H_2_O	60 °C; 15–45 min	5.02 ± 0.32; 2.53 ± 0.12 (µg GAE mg^−1^ extract)	[60]
blackberry (leaves)	20–80% MeOH; 0.4–1.6 M HCl	30–70 °C; 20–120 min	77.65	[62]
raspberry (leaves)	MeOH + 0.1% HCl	room temp.; 1 h	5.56 ± 0.058 mg g^−1^ dw	[64]
*Vaccinium* spp. (leaves)	85% MeOH + 0.5% formic acid	room temp.; 20 min	32.18–200.5	[71]
*Rubus idaeus* var. Tulameen (leaves)	60% EtOH	30 min	40.9 mg g^−1^ dw	[67]
*Rubus* cv. Loch Ness (leaves)	MeOH	room temp.; 30 min		[68]
*Ribes nigrum* L. (leaves)	40, 80% EtOH	20 °C; 55 min	39.96 ± 5.70	[70]
blackberry (leaves)	H_2_O; 70% MeOH	50 °C; 3 × 30 min	101.31 ± 0.11	[35]
*Rubus idaeus* (leaves)	80% MeOH	30 °C; 10 min	151.75 ± 20.67 mg 100 g^−1^ fw	[37]
blueberry (pomace)	acidified EtOH (fumaric, lactic, malic, and citric acid)	30, 35, 40 °C; 20, 30, 40 min; 300, 350, 400 W	108.23 mg/100 g dw	[72]
*Vaccinium ashei* Reade (pomace)	60% EtOH + 12 M HCl (*v*/*v* = 99/1)	300 W; 28 KHz; 1 h	57.35 ± 7.68%	[72]
blueberry (leaves)	40% EtOH	20 °C; 30 min	155.67 mg g^−1^	[69]
*Aronia melanocarpa* (pomace)	50% EtOH; 1% formic acid in 50% EtOH;	25, 50, 75% amplitude; 4, 6, 8, 10 min;	567.3 ± 2.4 mg 100 g^−1^ (total anthocyanin)	[52]
black currant and chokeberry (pomace)	H_2_O	30, 55, 80% amplitude; 2, 6 and 10 min		[53]
MAE				
*Aronia melanocarpa* (pomace)	1% formic acid in 50% EtOH; 50% EtOH	40, 60, 80 °C; 4, 6, 8, 10 min	552.6 ± 1.0 mg 100 g^−1^ (total anthocyanin)	[52]
*Vaccinium ashei* Reade (pomace)	60% EtOH + 12 M HCl (*v*/*v* = 99/1)	360 W; 150 s	55.07 ± 3.32%	[72]
*Vaccinium corymbosum* (pomace)	60% EtOH	30–70 °C; 2.45 ± 0.05 GHz; 1600 W	231.56 ± 4.41 mg 100 g^−1^ (total anthocyanin)	[73]
*Rubus Coreanus* Miq. (pomace)	EtOH, MeOH, acetone	3–9 min; 60–300 W	38.1	[74]
strawberry (leaves)	20–80% EtOH	10–60 s; 100–600 W; 1–4 extraction cycles	87.6	[75]
blueberry (leaves)	15% EtOH, 30% EtOH; 1.5 M citric acid	4, 10, 16 min; 10–20% absolute power	92.719–128.76	[76]
*Vaccinium corymbosum* (leaves)	30% EtOH + 1.5 M citric acid	24 min; 20% power	156.861 ± 3.203	[77]
*Vaccinium corymbosum* L. (leaves)	30% EtOH + 1.5 M citric acid	24 min; 20% power	161.709 ± 8.286	[78]
*Morus alba* L. (leaves)	deep eutectic solvent (DES)	60 °C; 600 W; 20 min	8.352 mg g^−1^	[79]
black chokeberry (leaves)	30% EtOH	60,70, 80 °C; 5, 10 min	23.9–49.9	[80]
*Ribes nigrum* L.; *Vaccinium myrtillus* L. (leaves)	30% EtOH	60,70, 80 °C; 5, 10 min	49.45–73.26; 34.32–64.19	[81]
PLE				
*Vaccinium myrtillus* L. (pomace)	acidified water (pH 2.0), 100% EtOH, 50% EtOH, 100% acetone	40 °C; 20 MPa; 10 mL/min	102 ± 1.0	[82]
black chokeberry (pomace)	hexane, MeOH, H_2_O, 80% MeOH, 80% acetone	40, 130 °C; 10.3 MPa; 45 min	48.13 ± 0.81 g 100 g^−1^ dw	[83]
cranberry (pomace)	H_2_O, 30, 70, 100% EtOH, 5% citric acid	40–160 °C; 50 and 200 bar	42.28 ± 7.82	[84]
*Fragaria vesca* L. (leaves)	30% EtOH	100, 125, 150 °C; 5 and 10 min	8027 mg GA 100 g^−1^ dw	[85]
*Fragaria* X *ananassa* (pomace)	EtOH; H_2_O	90, 110 °C; 10.3 MPa; 3 cycles × 15 min	21.5–29.6	[54]
*Vaccinium* spp. (pomace)	EtOH; MeOH; ethylene glycol 20%; propylene glycol 20%	40 °C; 100 bar; solvent flow rate 2 mL min^−1^; static extraction 30 min, dynamic extraction 180 min	3541.62–4116.62 mg GAE 100 g^−1^ dw	[86]
black chokeberry (leaves)	30% EtOH	100,125, 150 °C; 5, 10 min	35.1 ± 0.2–64.7 ± 0.5	[80]
*Ribes nigrum* L.; *Vaccinium myrtillus* L. (leaves)	30% EtOH	100,125, 150 °C; 5, 10 min	52.76–78.90; 33.74–70.55	[81]
HPE				
blueberry (pomace)	acetone/water/acetic acid (70/29.5/0.5)	room temp.; 500 MPa; 5, 10, 15 min	96.7–117.1	[87]
*Vaccinium ashei* Reade (pomace)	60% EtOH + 12 M HCl (*v*/*v* = 99/1)	room temp.; 500 MPa; 3 min	70.26 ± 5.63%	[72]
*Vaccinium ashei* (pomace)	20–80% EtOH	room temp.; 100–600 MPa; 5–30 min; 1–3 extraction cycles	85.14–108.76 mg 100 g^−1^ (anthocyanin content)	[88]
SFE				
*Vaccinium myrtillus* L. (pomace)	CO_2_ (100, 90, 50%), water (5, 10, 50%), acidified water (pH 2) (4, 8, 10, 50%), EtOH (1, 2, 5, 10, 50%)	15, 20, 25 MPa; CO_2_ flow rate: 1.05 × 10^−4^ and 1.4 × 10^−4^ kg/s	28–134	[82]
black chokeberry	2.5, 10% EtOH	149 min; 40 MPa; 40 °C; CO_2_ flow rate 2 L min^−1^	2.95–7.08 g 100 g^−1^ dw	[83]
*Arbutus unedo* L.	0, 10, 20% EtOH	40, 60, 80 °C; 150, 250, 350 bar; CO_2_ flow rate 15 g min^−1^	6.29–25.30	[89]
*Aronia melanocarpa* (pomace)	20, 50, 80% EtOH	35, 50, 65 °C; 7.5, 10, 12.5 MPa; CO_2_ flow rate 1.8 g min^−1^	187.2–1520.7 mg GA100 g^−1^	[90]

**Table 2:** Antioxidant capacity of pomace from selected berries (according to Struck et al. [111]).

Berries	DPPH	ABTS	Reference
Chokeberry (*Aronia melanocarpa* Elliot)	3.01 ^a^	7.79 ^a^	[115]
Chokeberry (*Aronia melanocarpa*)	-	240–600 ^b^	[116]
Chokeberry (*Aronia melanocarpa*)	100.8 ^b^	198.4 ^b^	[117]
Blueberry (*Vaccinium* spp.)	919.71 ^c^	122.56 ^a^	[118]
Raspberry (*Rubus idaeus*, cv. ‘Meeker’)	-	30.89 ^d^	[119]
Blackberry (*Rubus fructicosus*, cv. ‘Thornfree’)	-	12.36 ^d^	[119]
Blackcurrant (seedless)	101.0 ^b^	-	[120]

DPPH–2,2-diphenyl-1-picrylhydrazyl method; ABTS–2,2′-azino-bis(3-ehylbenzothiazoline-6-sulphonic acid) method. ^a^ µmol Trolox equivalents (TE)/g dry mass; ^b^ µmol TE/g wet mass; ^c^ g DPPH/g dry mass; ^d^ EC_50_ in mmol TE/g dry mass.

**Table 3:** Medicinal use and biological activity of the leaves of selected berries (according to Ferlemi and Lamari, [17]).

	European Medicines Agency	Traditional Use	In-Vitro/In-Vivo	Clinical Trials
Blackcurrant	TMP: Minor articular pain Adjuvant in minor urinary complaints [143]	Diaphoretic and diuretic agent Against diarrhea Against spasmodic cough Relief of rheumatic pain [138,143]	Antioxidant, Anti-inflammatory activity [134,143] Analgesic activity [138]	
Raspberry	TMP: Symptomatic relief of minor spasm associated with menstrual periods Symptomatic treatment of mild inflammation in the mouth or throat Symptomatic treatment of mild diarrhea [135]	Labor stimulator [152] Relief of menstrual cramps Relief of diarrhea Astringent agent Anti-inflammatory agent (mouth, throat) Against chronic skin conditions Treatment of conjunctivitis [153]	Antioxidant activity [134,138]	Indications that it facilitates labor [36,141,144]
Blackberry		Mouthwash against thrush, gum inflammations, mouth ulcers, sore throat Against respiratory problems Astringent agent Regulation of anemia, diarrhea, dysentery, cystitis, hemorrhoids [40]	Antidiabetic/Hypoglycemic activity [42,142,154] Antimicrobial activity [136] Analgesic, Anti-inflammatory, Angiogenic Activity [40,155,156]	
Bilberry		Diuretic, astringent and antiseptic agent for the urinary tract Antibacterial Anti-inflammatory Antidiabetic [38]	Antidiabetic activity [41,157,158] Anti-hyperlipidemic activity [159] Antistaphylococcal activity [160,161] Antioxidant, Anti-neoplastic activity [162]	
Cranberry			Antioxidant activity [162]	Antimicrobial agent—urinary tract protection Antioxidant activity [163]

**Table 4:** Overview of applied techniques and carriers for the encapsulation of berry pomace and leaf extracts.

Extract	Method	Carrier	Properties	Possible Application	References
Blueberry pomace extract	Ionic gelling	Na-alginate + ZnCl_2_	Retention of 67.01% of TPC	Incorporation into functional beverages, bakery and confectionery products	[176]
	Spray drying	Maltodextrin	High process utilization, powders with moisture content of ≈5%	Natural dyes in the food industry	[174]
	Spray drying	Whey protein isolate	5% moisture content in microcapsules, increased stability during storage	Food supplement	[175]
Chokeberry pomace extract	Emulsification + Freeze-drying	Oil phase: polyglycerol polyricinoleate + rapeseed oil Aqueous phase: NaCl, milk proteins	Encapsulation efficiency 95.36%, intense color, low moisture content	Confectionery industry	[178]
unprocessed aronia fruits extract	Spray drying/Co-crystallization/Ionic gelling	Maltodextrin, skimmed milk/Sucrose/Na-alginate + CaCl_2_	High efficiency of all encapsulation techniques, the highest stability of phenolic compounds during storage by spray drying encapsulation	Natural antioxidant in the food industry	[177]
Blueberry leaf extract	Nanoprecipitation	Eudragit^®^ RS 100	Increased ORAC value of the extract 2 folds, action similar to control antidepressants	Natural antidepressant	[179]
Bilberry, lingonberry, and blueberry leaf extracts	Spray drying	Maltodextrin + glucose	Encapsulation efficiency 79–81%, increased bioavailability of phenolic compounds	Functional supplements (nutraceuticals)	[180]
Mulberry leaf extract	Freeze-drying	Maltodextrin/Carboxymethyl cellulose	91.35–95.79% retention of phenolic acids with maltodextrin, 80.82–97.83% retention of flavonols with carboxymethylcellulose	Functional products; nutraceuticals	[182]
	Freeze-drying	Maltodextrin/Carboxymethyl cellulose	80.39–91.13% retention of phenolic acids with maltodextrin, 72.18–93.95% retention of flavonols with carboxymethylcellulose	Functional products and nutraceuticals	[181]
Goji leaf extract (*Lycium barbarum*)	Encapsulation in liposomes	Phosphatidylcholine/Phosphatidylcholine + Na-choline	Encapsulation efficiency 75–85%, cytoprotective effect on mouse fibroblast cells	Natural antioxidant	[183]

TPC—total phenolic content.

## Data Availability

No new data were created or analyzed in this study. Data sharing is not applicable to this article.

## References

[1] Eurostat (2023). Agricultural Production—Crops. https://ec.europa.eu/eurostat/statistics-explained/index.php?title=Agricultural_production_-_crops#Fruit.

[2] Mezzetti B. (2016). The sustainable improvement of European berry production, quality and nutritional value in a changing environment: Strawberries, currants, blackberries, blueberries and raspberries—The EUBerry project. Acta Hortic..

[3] Yang B., Kortesniemi M. (2015). Clinical evidence on potential health benefits of berries. Curr. Opin. Food Sci..

[4] Meremäe K., Raudsepp P., Rusalepp L., Anton D., Bleive U., Roasto M. (2024). In Vitro Antibacterial and Antioxidative Activity and Polyphenolic Profile of the Extracts of Chokeberry, Blackcurrant, and Rowan Berries and Their Pomaces. Foods.

[5] Skrovankova S., Sumczynski D., Mlcek J., Jurikova T., Sochor J. (2015). Bioactive Compounds and Antioxidant Activity in Different Types of Berries. Int. J. Mol. Sci..

[6] Kruczek M., Drygaś B., Habryka C. (2016). Pomace in fruit industry and their contemporary potential application. World Sci. News.

[7] Majerska J., Michalska A., Figiel A. (2019). A review of new directions in managing fruit and vegetable processing by-products. Trends Food Sci. Technol..

[8] Chaouch M.A., Benvenuti S. (2020). The Role of Fruit by-Products as Bioactive Compounds for Intestinal Health. Foods.

[9] May N., Guenther E. (2020). Shared benefit by Material Flow Cost Accounting in the food supply chain—The case of berry pomace as upcycled by-product of a black currant juice production. J. Clean. Prod..

[10] Sobhy R., Öz F., Lorenzo J.M., Bakry A.M., Mohamed A., Khalifa I., Nawaz A. (2023). Bioactive components and health promoting effect of berry by-products. Berry Bioactive Compound By-Products.

[11] El Maaiden E., Bouzroud S., Nasser B., Moustaid K., El Mouttaqi A., Ibourki M., Boukcim H., Hirich A., Kouisni L., El Kharrassi Y. (2022). A Comparative Study between Conventional and Advanced Extraction Techniques: Pharmaceutical and Cosmetic Properties of Plant Extracts. Molecules.

[12] Nirmal N.P., Khanashyam A.C., Mundanat A.S., Shah K.R., Babu K.S., Thorakkattu P., Al-Asmari F., Pandiselvam R. (2023). Valorization of Fruit Waste for Bioactive Compounds and Their Applications in the Food Industry. Foods.

[13] Alu’datt M.H., Alrosan M., Gammoh S., Tranchant C.C., Alhamad M.N., Rababah T., Zghoul R., Alzoubi H., Ghatasheh S., Ghozlan K. (2022). Encapsulation-based technologies for bioactive compounds and their application in the food industry: A roadmap for food-derived functional and health-promoting ingredients. Food Biosci..

[14] Jiang Y., Subbiah V., Wu H., Bk A., Sharifi-Rad J., Suleria H.A.R. (2022). Phenolic Profiling of Berries Waste and Determination of Their Antioxidant Potential. J. Food Qual..

[15] Dukare A.S., Paul S., Nambi V.E., Gupta R.K., Singh R., Sharma K., Vishwakarma R.K. (2019). Exploitation of microbial antagonists for the control of postharvest diseases of fruits: A review. Crit. Rev. Food Sci. Nutr..

[16] Klavins L., Kviesis J., Nakurte I., Klavins M. (2018). Berry press residues as a valuable source of polyphenolics: Extraction optimisation and analysis. LWT.

[17] Ferlemi A.V., Lamari F.N. (2016). Berry Leaves: An Alternative Source of Bioactive Natural Products of Nutritional and Medicinal Value. Antioxidants.

[18] Maatta-Riihinen K.R., Kamal-Eldin A., Torronen A.R. (2004). Identification and quantification of phenolic compounds in berries of *Fragaria* and *Rubus* species (Family rosaceae). J. Agric. Food Chem..

[19] Suvanto J., Karppinen K., Riihinen K., Jaakola L., Salminen J.P. (2020). Changes in the Proanthocyanidin Composition and Related Gene Expression in Bilberry (*Vaccinium myrtillus* L.) Tissues. J. Agric. Food Chem..

[20] Yan Y.F., Pico J., Sun B.H., Pratap-Singh A., Gerbrandt E., Castellarin S.D. (2023). Phenolic profiles and their responses to pre- and post-harvest factors in small fruits: A review. Crit. Rev. Food Sci. Nutr..

[21] Bouyahya A., El Omari N., El Hachlafi N., El Jemly M., Hakkour M., Balahbib A., El Menyiy N., Bakrim S., Mrabti H.N., Khouchlaa A. (2022). Chemical Compounds of Berry-Derived Polyphenols and Their Effects on Gut Microbiota, Inflammation, and Cancer. Molecules.

[22] Bai X.Y., Zhou L., Zhou L., Cang S., Liu Y.H., Liu R., Liu J., Feng X., Fan R.H. (2023). The Research Progress of Extraction, Purification and Analysis Methods of Phenolic Compounds from Blueberry: A Comprehensive Review. Molecules.

[23] Shi M., Loftus H., McAinch A.J., Su X.Q. (2017). Blueberry as a source of bioactive compounds for the treatment of obesity, type 2 diabetes and chronic inflammation. J. Funct. Foods.

[24] Del Rio D., Borges G., Crozier A. (2010). Berry flavonoids and phenolics: Bioavailability and evidence of protective effects. Br. J. Nutr..

[25] Vendrame S., Del Bo’ C., Ciappellano S., Riso P., Klimis-Zacas D. (2016). Berry Fruit Consumption and Metabolic Syndrome. Antioxidants.

[26] Olas B. (2018). The beneficial health aspects of sea buckthorn (*Elaeagnus rhamnoides* (L.) A.Nelson) oil. J. Ethnopharmacol..

[27] Bernjak B., Kristl J. (2021). A Review of Tannins in Berries. Agric. Sci..

[28] Teleszko M., Wojdylo A. (2015). Comparison of phenolic compounds and antioxidant potential between selected edible fruits and their leaves. J. Funct. Foods.

[29] Diaconeasa Z., Leopold L., Rugina D., Ayvaz H., Socaciu C. (2015). Antiproliferative and Antioxidant Properties of Anthocyanin Rich Extracts from Blueberry and Blackcurrant Juice. Int. J. Mol. Sci..

[30] Rocchetti G., Gregorio R.P., Lorenzo J.M., Barba F.J., Oliveira P.G., Prieto M.A., Simal-Gandara J., Mosele J.I., Motilva M.-J., Tomas M. (2022). Functional implications of bound phenolic compounds and phenolics–food interaction: A review. Compr. Rev. Food Sci. Food Saf..

[31] Okatan V., Gündoğdu M., Güçlü S.F., Çelikay Özaydın A., Çolak A.M., Korkmaz N., Polat M., Çelik F., Aşkın M.A. (2017). Phenolic Profiles of Currant (*Ribes* spp.) Cultivars. Yuz. Yıl Univ. J. Agric. Sci..

[32] Häkkinen S.H., Kärenlampi S.O., Mykkänen H.M., Heinonen I.M., Törrönen A.R. (2000). Ellagic acid content in berries: Influence of domestic processing and storage. Eur. Food Res. Technol..

[33] Vrhovsek U., Masuero D., Palmieri L., Mattivi F. (2012). Identification and quantification of flavonol glycosides in cultivated blueberry cultivars. J. Food Compos. Anal..

[34] Varzaru I., Oancea A.G., Vlaicu P.A., Saracila M., Untea A.E. (2023). Exploring the Antioxidant Potential of Blackberry and Raspberry Leaves: Phytochemical Analysis, Scavenging Activity, and In Vitro Polyphenol Bioaccessibility. Antioxidants.

[35] Paczkowska-Walendowska M., Gościniak A., Szymanowska D., Szwajgier D., Baranowska-Wójcik E., Szulc P., Dreczka D., Simon M., Cielecka-Piontek J. (2021). Blackberry Leaves as New Functional Food? Screening Antioxidant, Anti-Inflammatory and Microbiological Activities in Correlation with Phytochemical Analysis. Antioxidants.

[36] Wang S.Y., Lin H.S. (2000). Antioxidant activity in fruits and leaves of blackberry, raspberry, and strawberry varies with cultivar and developmental stage. J. Agric. Food Chem..

[37] Ponder A., Hallmann E. (2019). Phenolics and Carotenoid Contents in the Leaves of Different Organic and Conventional Raspberry (*Rubus idaeus* L.) Cultivars and Their In Vitro Activity. Antioxidants.

[38] Gudej J. (2003). Kaempferol and quercetin glycosides from *Rubus idaeus* L. leaves. Acta Pol. Pharm..

[39] Ieri F., Martini S., Innocenti M., Mulinacci N. (2013). Phenolic Distribution in Liquid Preparations of *Vaccinium myrtillus* L. and *Vaccinium vitis idaea* L.. Phytochem. Anal..

[40] Oszmianski J., Wojdylo A., Gorzelany J., Kapusta I. (2011). Identification and Characterization of Low Molecular Weight Polyphenols in Berry Leaf Extracts by HPLC-DAD and LC-ESI/MS. J. Agric. Food Chem..

[41] Riihinen K., Jaakola L., Kärenlampi S., Hohtola A. (2008). Organ-specific distribution of phenolic compounds in bilberry (*Vaccinium myrtillus*) and ‘northblue’ blueberry (*Vaccinium corymbosum* x *V. angustifolium*). Food Chem..

[42] Oszmianski J., Wojdylo A., Nowicka P., Teleszko M., Cebulak T., Wolanin M. (2015). Determination of Phenolic Compounds and Antioxidant Activity in Leaves from Wild *Rubus* L. Species. Molecules.

[43] Vagiri M., Conner S., Stewart D., Andersson S.C., Verrall S., Johansson E., Rumpunen K. (2015). Phenolic compounds in blackcurrant (*Ribes nigrum* L.) leaves relative to leaf position and harvest date. Food Chem..

[44] Martynenko K., Balanov P., Smotraeva I., Vanchenko O.I. (2022). Exploring the potential of total polyphenols in the leaves of berry plants for a healthy diet. BIO Web Conf..

[45] Huang H.-W., Hsu C.-P., Yang B.B., Wang C.Y. (2013). Advances in the extraction of natural ingredients by high pressure extraction technology. Trends Food Sci. Technol..

[46] Belwal T., Ezzat S.M., Rastrelli L., Bhatt I.D., Daglia M., Baldi A., Devkota H.P., Orhan I.E., Patra J.K., Das G. (2018). A critical analysis of extraction techniques used for botanicals: Trends, priorities, industrial uses and optimization strategies. Trends Anal. Chem..

[47] Bamba B.S.B., Shi J., Tranchant C.C., Xue S.J., Forney C.F., Lim L.T. (2018). Influence of Extraction Conditions on Ultrasound-Assisted Recovery of Bioactive Phenolics from Blueberry Pomace and Their Antioxidant Activity. Molecules.

[48] Patra A., Abdullah S., Pradhan R.C. (2022). Review on the extraction of bioactive compounds and characterization of fruit industry by-products. Bioresour. Bioprocess..

[49] He B., Zhang L.L., Yue X.Y., Liang J., Jiang J., Gao X.L., Yue P.X. (2016). Optimization of Ultrasound-Assisted Extraction of phenolic compounds and anthocyanins from blueberry (*Vaccinium ashei*) wine pomace. Food Chem..

[50] dos Santos S.S., Paraíso C.M., Romanini E.B., Correa V.G., Peralta R.M., da Costa S.C., Santos O.D., Visentainer J.V., Reis M.H.M., Madrona G.S. (2022). Bioavailability of blackberry pomace microcapsules by using different techniques: An approach for yogurt application. Innov. Food Sci. Emerg. Technol..

[51] Piasecka I., Brzezińska R., Kalisz S., Wiktor A., Górska A. (2024). Response Surface Methodology for Optimization of Ultrasound-Assisted Antioxidants Extraction from Blackberry, Chokeberry and Raspberry Pomaces. Plants.

[52] Garofulic I.E., Repajic M., Zoric Z., Jurendic T., Dragovic-Uzelac V. (2023). Evaluation of Microwave- and Ultrasound-Assisted Extraction Techniques for Revalorization of Black Chokeberry (*Aronia melanocarpa*) Fruit Pomace Anthocyanins. Sustainability.

[53] Piasecka I., Górska A., Kalisz S., Brzezińska R., Wiktor A. (2022). Ultrasound-Assisted Extraction of Bioactive Compounds from Black Currant and Chokeberry Pomaces. Biol. Life Sci. Forum.

[54] Pukalskienė M., Pukalskas A., Dienaitė L., Revinytė S., Pereira C.V., Matias A.A., Venskutonis P.R. (2021). Recovery of Bioactive Compounds from Strawberry (*Fragaria* × *ananassa*) Pomace by Conventional and Pressurized Liquid Extraction and Assessment Their Bioactivity in Human Cell Cultures. Foods.

[55] Brnčić M., Tripalo B., Penava A., Karlović D., Ježek D., Vikić Topić D., Karlović S., Bosiljkov T. (2009). Primjena ultrazvuka visokog intenziteta pri obradi hrane. Croat. J. Food Technol. Biotechnol. Nutr..

[56] Kruszewski B., Boselli E. (2024). Blackcurrant Pomace as a Rich Source of Anthocyanins: Ultrasound-Assisted Extraction under Different Parameters. Appl. Sci..

[57] Alara O.R., Abdurahman N.H., Ukaegbu C.I. (2021). Extraction of phenolic compounds: A review. Curr. Res. Food Sci..

[58] Awad T.S., Moharram H.A., Shaltout O.E., Asker D., Youssef M.M. (2012). Applications of ultrasound in analysis, processing and quality control of food: A review. Food Res. Int..

[59] Ahmad-Qasem M.H., Cánovas J.S., Barrajón-Catalán E., Micol V., Cárcel J.A., García-Pérez J.V. (2013). Kinetic and compositional study of phenolic extraction from olive leaves (var. Serrana) by using power ultrasound. Innov. Food Sci. Emerg. Technol..

[60] dos Santos S.S., Paraíso C.M., Rodrigues L.M., Madrona G.S. (2021). Agro-industrial waste as a source of bioactive compounds: Ultrasound-assisted extraction from blueberry (*Vaccinium myrtillus*) and raspberry (*Rubus idaeus*) pomace. Acta Sci.-Technol..

[61] Loncaric A., Celeiro M., Jozinovic A., Jelinic J., Kovac T., Jokic S., Babic J., Moslavac T., Zavadlav S., Lores M. (2020). Green Extraction Methods for Extraction of Polyphenolic Compounds from Blueberry Pomace. Foods.

[62] Aybastier Ö., Isik E., Sahin S., Demir C. (2013). Optimization of ultrasonic-assisted extraction of antioxidant compounds from blackberry leaves using response surface methodology. Ind. Crops Prod..

[63] Pavlovic A.V., Papetti A., Dabic Zagorac D., Gasic U.M., Misic D.M., Tesic Z.L., Natic M.M. (2016). Phenolics composition of leaf extracts of raspberry and blackberry cultivars grown in Serbia. Ind. Crops Prod..

[64] Luo T., Chen S., Zhang H., Jia S., Wang J. (2020). Phytochemical composition and potential biological activities assessment of raspberry leaf extracts from nine different raspberry species and raspberry leaf tea. J. Berry Res..

[65] Zhang W.T., Xu S.J., Gao M., Peng S.J., Chen L.Y., Lao F., Liao X.J., Wu J.H. (2022). Profiling the water soluble pectin in clear red raspberry (*Rubus idaeus* L. cv. Heritage) juice: Impact of high hydrostatic pressure and high-temperature short-time processing on the pectin properties. Food Hydrocolloid.

[66] Wu L.Y., Yang J., Wang C.Y., Li N.A., Liu Y.P., Duan A.B., Wang T. (2022). Chemical compositions of raspberry leaves influenced by growth season, cultivars and leaf position. Sci. Hortic..

[67] Yang Y.J., Yin X.X., Zhang D.J., Zhang B.Y., Lu J., Wang X.H. (2022). Structural Characteristics, Antioxidant, and Immunostimulatory Activities of an Acidic Polysaccharide from Raspberry Pulp. Molecules.

[68] Gradillas A., Martínez-Alcázar M.P., Gutiérrez E., Ramos-Solano B., García A. (2019). A novel strategy for rapid screening of the complex triterpene saponin mixture present in the methanolic extract of blackberry leaves (*Rubus* cv. Loch Ness) by UHPLC/QTOF-MS. J. Pharm. Biomed. Anal..

[69] Stefanescu B.E., Calinoiu L.F., Ranga F., Fetea F., Mocan A., Vodnar D.C., Crisan G. (2020). The Chemical and Biological Profiles of Leaves from Commercial Blueberry Varieties. Plants.

[70] Nour V., Trandafir I., Cosmulescu S. (2014). Antioxidant capacity, phenolic compounds and minerals content of blackcurrant (*Ribes nigrum* L.) leaves as influenced by harvesting date and extraction method. Ind. Crops Prod..

[71] Wu H., Chai Z., Hutabarat R.P., Zeng Q.L., Niu L.Y., Li D.J., Yu H., Huang W.Y. (2019). Blueberry leaves from 73 different cultivars in southeastern China as nutraceutical supplements rich in antioxidants. Food Res. Int..

[72] Zhang H.N., Tchabo W., Ma Y.K. (2017). Quality of extracts from blueberry pomace by high hydrostatic pressure, ultrasonic, microwave and heating extraction: A comparison study. Emir. J. Food Agric..

[73] Liu C.H., Zhao X.L., Wang Z., Zheng X.Z., Xue L.L., Liu C., Chen Z.Y., Zhang B.F. (2022). Evaluation of the antioxidant activity of blueberry ethanol extracts under microwave extraction. Int. J. Agric. Biol. Eng..

[74] Teng H., Lee W.Y., Choi Y.H. (2013). Optimization of microwave-assisted extraction for anthocyanins, polyphenols, and antioxidants from raspberry (*Rubus Coreanus* Miq.) using response surface methodology. J. Sep. Sci..

[75] Lin D., Ma Q., Zhang Y., Peng Z. (2020). Phenolic compounds with antioxidant activity from strawberry leaves: A study on microwave-assisted extraction optimization. Prep. Biochem. Biotechnol..

[76] Routray W., Orsat V. (2014). MAE of phenolic compounds from blueberry leaves and comparison with other extraction methods. Ind. Crops Prod..

[77] Routray W., Orsat V. (2014). Variation of phenolic profile and antioxidant activity of North American highbush blueberry leaves with variation of time of harvest and cultivar. Ind. Crops Prod..

[78] Routray W., Orsat V., Lefsrud M. (2018). Effect of Postharvest LED Application on Phenolic and Antioxidant Components of Blueberry Leaves. Chemengineering.

[79] Gao M.-Z., Cui Q., Wang L.-T., Meng Y., Yu L., Li Y.-Y., Fu Y.-J. (2020). A green and integrated strategy for enhanced phenolic compounds extraction from mulberry (*Morus alba* L.) leaves by deep eutectic solvent. Microchem. J..

[80] Repajić M., Elez Garofulić I., Cegledi E., Dobroslavić E., Pedisić S., Durgo K., Huđek Turković A., Mrvčić J., Hanousek Čiča K., Dragović-Uzelac V. (2024). Bioactive and Biological Potential of Black Chokeberry Leaves Under the Influence of Pressurized Liquid Extraction and Microwave-Assisted Extraction. Antioxidants.

[81] Garofulic I.E., Repajic M., Cegledi E., Dobroslavic E., Dobrincic A., Zoric Z., Pedisic S., Frankovic T., Breski M., Dragovic-Uzelac V. (2024). Green Approach to Enhance the Recovery of Polyphenols from Blackcurrant and Bilberry Leaves: Evaluation of Microwave-Assisted and Pressurized Liquid Extraction. Molecules.

[82] Paes J.L., Dotta R., Barbero G.F., Martínez J. (2014). Extraction of phenolic compounds and anthocyanins from blueberry (*Vaccinium myrtillus* L.) residues using supercritical CO_2_ and pressurized liquids. J. Supercrit. Fluids.

[83] Grunovaitė L., Pukalskienė M., Pukalskas A., Venskutonis P.R. (2016). Fractionation of black chokeberry pomace into functional ingredients using high pressure extraction methods and evaluation of their antioxidant capacity and chemical composition. J. Funct. Foods.

[84] Saldaña M.D.A., Martinez E.R., Sekhon J.K., Vo H. (2021). The effect of different pressurized fluids on the extraction of anthocyanins and total phenolics from cranberry pomace. J. Supercrit. Fluids.

[85] Terpinc P., Dobroslavic E., Garofulic I.E., Repajic M., Cegledi E., Dobrincic A., Pedisic S., Levaj B. (2023). Maximizing the Recovery of Phenolic Antioxidants from Wild Strawberry (*Fragaria vesca*) Leaves Using Microwave-Assisted Extraction and Accelerated Solvent Extraction. Processes.

[86] Secco M.C., Fischer B., Fernandes I.A., Cansian R.L., Paroul N., Junges A. (2022). Valorization of Blueberry By-Products (*Vaccinium* spp.): Antioxidants by Pressurized Liquid Extraction (PLE) and Kinetics Models. Biointerface Res. Appl. Chem..

[87] Briones-Labarca V., Giovagnoli-Vicuña C., Chacana-Ojeda M. (2019). High pressure extraction increases the antioxidant potential and in vitro bio-accessibility of bioactive compounds from discarded blueberries. CyTA—J. Food.

[88] Zhang H.N., Ma Y.K. (2017). Optimisation of High Hydrostatic Pressure Assisted Extraction of Anthocyanins from Rabbiteye Blueberry Pomace. Czech J. Food Sci..

[89] Akay S., Alpak I., Yesil-Celiktas O. (2011). Effects of process parameters on supercritical CO_2_ extraction of total phenols from strawberry (*Arbutus unedo* L.) fruits: An optimization study. J. Sep. Sci..

[90] Wozniak L., Marszalek K., Skapska S., Jedrzejczak R. (2017). The Application of Supercritical Carbon Dioxide and Ethanol for the Extraction of Phenolic Compounds from Chokeberry Pomace. Appl. Sci..

[91] Veggi P.C., Martinez J., Meireles M.A.A., Chemat F., Cravotto G. (2013). Fundamentals of Microwave Extraction. Microwave-Assisted Extraction for Bioactive Compounds: Theory and Practice.

[92] Blekić M., Režek Jambrak A., Chemat F. (2011). Mikrovalna ekstrakcija bioaktivnih spojeva. Croat. J. Food Sci. Technol..

[93] Chan C.H., Yusoff R., Ngoh G.C., Kung F.W.L. (2011). Microwave-assisted extractions of active ingredients from plants. J. Chromatogr. A.

[94] Routray W., Orsat V. (2012). Microwave-Assisted Extraction of Flavonoids: A Review. Food Bioprocess Technol..

[95] Routray W., Orsat V., Gariepy Y. (2014). Effect of Different Drying Methods on the Microwave Extraction of Phenolic Components and Antioxidant Activity of Highbush Blueberry Leaves. Dry. Technol..

[96] Maccarronello E.A., Cardullo N., Margarida Silva A., Di Francesco A., Costa P.C., Rodrigues F., Muccilli V. (2024). From waste to bioactive compounds: A response surface methodology approach to extract antioxidants from *Pistacia vera* shells for postprandial hyperglycaemia management. Food Chem..

[97] Bouras M., Chadni M., Barba F.J., Grimi N., Bals O., Vorobiev E. (2015). Optimization of microwave-assisted extraction of polyphenols from *Quercus* bark. Ind. Crops Prod..

[98] Jentzer J.B., Alignan M., Vaca-Garcia C., Rigal L., Vilarem G. (2015). Response surface methodology to optimise Accelerated Solvent Extraction of steviol glycosides from *Stevia rebaudiana* Bertoni leaves. Food Chem..

[99] Mottaleb M.A., Sarker S.D., Sarker S.D., Nahar L. (2012). Accelerated Solvent Extraction for Natural Products Isolation. Natural Products Isolation, Methods in Molecular Biology.

[100] Ray A., Dubey K.K., Marathe S.J., Singhal R. (2023). Supercritical fluid extraction of bioactives from fruit waste and its therapeutic potential. Food Biosci..

[101] Jokic S. (2011). Matematičko Modeliranje Ekstrakcije Ulja iz Zrna soje Superkritičnim CO_2_. Ph.D. Thesis.

[102] Punvichai T., Pipakdee J., Chaitham H., Kritsanapuntu S., Chotimarkorn C., Detarun P. (2024). Factors affecting the quality of supercritical carbon dioxide extract from *Curcuma longa* Linn in Thailand: Antioxidant and lipid composition. Food Chem. Adv..

[103] Uwineza P.A., Waśkiewicz A. (2020). Recent Advances in Supercritical Fluid Extraction of Natural Bioactive Compounds from Natural Plant Materials. Molecules.

[104] Morata A., Del Fresno J.M., Gavahian M., Guamis B., Palomero F., López C. (2023). Effect of HHP and UHPH High-Pressure Techniques on the Extraction and Stability of Grape and Other Fruit Anthocyanins. Antioxidants.

[105] Cai H., You S., Xu Z., Li Z., Guo J., Ren Z., Fu C. (2021). Novel extraction methods and potential applications of polyphenols in fruit waste: A review. J. Food Meas. Charact..

[106] Ferro D.M., Mayer D.A., Oliveira Müller C.M., Ferreira S.R.S. (2020). Scale-up simulation of PLE process applied to recover bio-based materials from *Sida rhombifolia* leaves. J. Supercrit. Fluids.

[107] Santos J., Santana L. (2022). Conventional and emerging techniques for extraction of bioactive compounds from fruit waste. Braz. J. Food Technol..

[108] Daglia M. (2012). Polyphenols as antimicrobial agents. Curr. Opin. Biotechnol..

[109] Ortega A.M.M., Campos M.R.S. (2019). Bioactive Compounds as Therapeutic Alternatives. Bioactive Compounds: Health Benefits and Potential Applications.

[110] Eseberri I., Trepiana J., Léniz A., Gómez-García I., Carr-Ugarte H., González M., Portillo M.P. (2022). Variability in the Beneficial Effects of Phenolic Compounds: A Review. Nutrients.

[111] Struck S., Plaza M., Turner C., Rohm H. (2016). Berry pomace—A review of processing and chemical analysis of its polyphenols. Int. J. Food Sci. Technol..

[112] Quirós-Sauceda A.E., Palafox-Carlos H., Sáyago-Ayerdi S.G., Ayala-Zavala J.F., Bello-Perez L.A., Alvarez-Parrilla E., de la Rosa L.A., González-Córdova A.F., González-Aguilar G.A. (2014). Dietary fiber and phenolic compounds as functional ingredients: Interaction and possible effect after ingestion. Food Funct..

[113] Piechowiak T., Skóra B., Grzelak-Błaszczyk K., Sójka M. (2021). Extraction of Antioxidant Compounds from Blueberry Fruit Waste and Evaluation of Their In Vitro Biological Activity in Human Keratinocytes (HaCaT). Food Anal. Methods.

[114] Alam M.N., Bristi N.J., Rafiquzzaman M. (2013). Review on in vivo and in vitro methods evaluation of antioxidant activity. Saudi Pharm. J..

[115] Oszmianski J., Wojdylo A. (2005). *Aronia melanocarpa* phenolics and their antioxidant activity. Eur. Food Res. Technol..

[116] Mayer-Miebach E., Adamiuk M., Behsnilian D. (2012). Stability of Chokeberry Bioactive Polyphenols during Juice Processing and Stabilization of a Polyphenol-Rich Material from the By-Product. Agriculture.

[117] Kapci B., Neradová E., Cízková H., Voldrich M., Rajchl A., Capanoglu E. (2013). Investigating the antioxidant potential of chokeberry (*Aronia melanocarpa*) products. J. Food Nutr. Res..

[118] Reque P.M., Stefens R.S., Da Silva A.M., Jablonski A., Flôres S.H., Rios A.D., De Jong E.V. (2014). Characterization of blueberry fruits (*Vaccinium* spp.) and derived products. J. Food Sci. Technol..

[119] Šaponjac V.T., Gironés-Vilaplana A., Djilas S., Mena P., Cetković G., Moreno D.A., Canadanović-Brunet J., Vulić J., Stajčić S., Krunić M. (2014). Anthocyanin profiles and biological properties of caneberry (*Rubus* spp.) press residues. J. Sci. Food Agric..

[120] Sójka M., Król B. (2009). Composition of industrial seedless black currant pomace. Eur. Food Res. Technol..

[121] Sarv V., Venskutonis P.R., Rätsep R., Aluvee A., Kazernavičiūtė R., Bhat R. (2021). Antioxidants Characterization of the Fruit, Juice, and Pomace of Sweet Rowanberry (*Sorbus aucuparia* L.) Cultivated in Estonia. Antioxidants.

[122] Zhang M.-Q., Zhang J., Zhang Y.-T., Sun J.-Y., Prieto M.A., Simal-Gandara J., Putnik P., Li N.-Y., Liu C. (2023). The link between the phenolic composition and the antioxidant activity in different small berries: A metabolomic approach. LWT.

[123] Aly A.A., Ali H.G.M., Eliwa N.E.R. (2019). Phytochemical screening, anthocyanins and antimicrobial activities in some berries fruits. J. Food Meas. Charact..

[124] Bartkiene E., Lele V., Sakiene V., Zavistanaviciute P., Ruzauskas M., Bernatoniene J., Jakstas V., Viskelis P., Zadeike D., Juodeikiene G. (2019). Improvement of the antimicrobial activity of lactic acid bacteria in combination with berries/fruits and dairy industry by-products. J. Sci. Food Agric..

[125] Das Q., Islam M.R., Marcone M.F., Warriner K., Diarra M.S. (2017). Potential of berry extracts to control foodborne pathogens. Food Control.

[126] Caillet S., Côté J., Sylvain J.F., Lacroix M. (2012). Antimicrobial effects of fractions from cranberry products on the growth of seven pathogenic bacteria. Food Control.

[127] Perumal S., Mahmud R., Ismail S. (2017). Mechanism of Action of Isolated Caffeic Acid and Epicatechin 3-gallate from *Euphorbia hirta* against *Pseudomonas aeruginosa*. Pharmacogn. Mag..

[128] De Rossi L., Rocchetti G., Lucini L., Rebecchi A. (2025). Antimicrobial Potential of Polyphenols: Mechanisms of Action and Microbial Responses—A Narrative Review. Antioxidants.

[129] Lau A.T.Y., Barbut S., Ross K., Diarra M.S., Balamurugan S. (2019). The effect of cranberry pomace ethanol extract on the growth of meat starter cultures, *Escherichia coli* O157:H7, *Salmonella enterica* serovar Enteritidis and *Listeria monocytogenes*. LWT.

[130] Ospina M., Montaña-Oviedo K., Díaz-Duque A., Toloza-Daza H., Narváez-Cuenca C.E. (2019). Utilization of fruit pomace, overripe fruit, and bush pruning residues from Andes berry (*Rubus glaucus* Benth) as antioxidants in an oil in water emulsion. Food Chem..

[131] Peiretti P.G., Gai F., Zorzi M., Aigotti R., Medana C. (2020). The effect of blueberry pomace on the oxidative stability and cooking properties of pork patties during chilled storage. J. Food Process. Preserv..

[132] Molan A.L., Lila M.A., Mawson J., De S. (2009). In vitro and in vivo evaluation of the prebiotic activity of water-soluble blueberry extracts. World J. Microbiol. Biotechnol..

[133] Lavefve L., Howard L.R., Carbonero F. (2020). Berry polyphenols metabolism and impact on human gut microbiota and health. Food Funct..

[134] Raudsepp P., Kaldmäe H., Kikas A., Libek A.V., Püssa T. (2010). Nutritional quality of berries and bioactive compounds in the leaves of black currant (*Ribes nigrum* L.) cultivars evaluated in Estonia. J. Berry Res..

[135] Rasheed H., Nawaz H., Rehman R., Mushtaq A., Rashid U. (2017). The Blackberry: A Review on Its Composition and Chemistry, Uses and Bioavailability and Potential Health Benefits. Int. J. Chem. Biol. Sci..

[136] Zia-Ul-Haq M., Riaz M., De Feo V., Jaafar H.Z., Moga M. (2014). *Rubus fruticosus* L.: Constituents, biological activities and health related uses. Molecules.

[137] Tabart J., Franck T., Kevers C., Pincemail J., Serteyn D., Defraigne J.O., Dornmes J. (2012). Antioxidant and anti-inflammatory activities of *Ribes nigrum* extracts. Food Chem..

[138] Committee on Herbal Medicinal Products (HMPC) (2012). Assessment Report on Rubus idaeus L., Folium.

[139] Garbacki N., Angenot L., Bassleer C., Damas J., Tits M. (2002). Effects of prodelphinidins isolated from *Ribes nigrum* on chondrocyte metabolism and COX activity. Naunyn-Schmiedeberg’s Arch. Pharmacol..

[140] Xu Y., Zhang Y., Chen M. (2006). Effective Fractions of *Rubus fruticosus* Leaf, Its Pharmaceutical Composition and Uses for Prevention and Treatment of Diabetes. Chinese Patent.

[141] Greenway F., Liu Z., Woltering E. (2010). Angiogenic Agents from Plant Extracts, Gallic Acid and Derivatives. U.S. Patent.

[142] Gudej J., Tomczyk M. (2004). Determination of flavonoids, tannins and ellagic acid in leaves from *Rubus* L. species. Arch. Pharmacal Res..

[143] Committee on Herbal Medicinal Products (HMPC) (2012). Community Herbal Monograph on Rubus idaeus L., Folium.

[144] Scientific Committee of the British Herbal Medicine Association (SCBHMA) (1983). British Herbal Pharmacopoeia 1983.

[145] Parsons M., Simpson M., Ponton T. (1999). Raspberry leaf and its effect on labour: Safety and efficacy. Aust. Coll. Midwives Inc. J..

[146] Cignarella A., Nastasi M., Cavalli E., Puglisi L. (1996). Novel lipid-lowering properties of *Vaccinium myrtillus* L. leaves, a traditional antidiabetic treatment, in several models of rat dyslipidaemia: A comparison with ciprofibrate. Thromb. Res..

[147] Petlevski R., Hadžija M., Slijepčević M., Juretić D. (2001). Effect of ‘antidiabetis’ herbal preparation on serum glucose and fructosamine in NOD mice. J. Ethnopharmacol..

[148] Sadowska B., Paszkiewicz M., Podsędek A., Redzynia M., Różalska B. (2014). *Vaccinium myrtillus* leaves and *Frangula alnus* bark derived extracts as potential antistaphylococcal agents. Acta Biochim. Pol..

[149] Mechikova G.Y., Kuzmich A.S., Ponomarenko L.P., Kalinovsky A.I., Stepanova T.A., Fedorov S.N., Stonik V.A. (2010). Cancer-preventive activities of secondary metabolites from leaves of the bilberry *Vaccinium smallii* A. Gray. Phytother. Res..

[150] Skupien K., Osmianski J., Kostrzewa-Nowak D., Tarasiuk J. (2006). In vitro antileukaemic activity of extracts from berry plant leaves against sensitive and multidrug resistant HL60 cells. Cancer Lett..

[151] Mudnic I., Modun D., Brizic I., Vukovic J., Generalic I., Katalinic V., Bilusic T., Ljubenkov I., Boban M. (2009). Cardiovascular effects in vitro of aqueous extract of wild strawberry (*Fragaria vesca* L.) leaves. Phytomedicine.

[152] Riaz M., Ahmad M., Rahaman N. (2011). Antimicrobial screening of fruit, leaves, root and stem of *Rubus fruticosus*. J. Med. Plants Res..

[153] Sarkar A., Ganguly S.N. (1978). Rubitic acid, a new triterpene acid from *Rubus fruticosus*. Phytochemistry.

[154] Abu-Shandi K., Al-Rawashdeh A., Al-Mazaideh G., Abu-Nameh E., Al-Amro A., Al-Soufi H., Al-Ma’abreh A., Al-Dawdeyah A. (2015). Novel strategy for the identification of the medicinal natural products in *Rubus fruticosus* plant by using GC/MS technique: A study on leaves, stems and roots of the plant. Adv. Anal. Chem..

[155] Mukherjee M., Ghatak K.L., Ganguly S.N., Antoulas S. (1984). Rubinic acid, a triterpene acid from *Rubus fruticosus*. Phytochemistry.

[156] Robinson G.M., Robinson R. (1932). A survey of anthocyanins. II. Biochem. J..

[157] Hokkanen J., Mattila S., Jaakola L., Pirttilä A.M., Tolonen A. (2009). Identification of phenolic compounds from lingonberry (*Vaccinium vitis-idaea* L.), bilberry (*Vaccinium myrtillus* L.) and hybrid bilberry (*Vaccinium x intermedium Ruthe* L.) leaves. J. Agric. Food Chem..

[158] Szakiel A., Pączkowski C., Huttunen S. (2012). Triterpenoid content of berries and leaves of bilberry *Vaccinium myrtillus* from Finland and Poland. J. Agric. Food Chem..

[159] Jaakola L., Määttä-Riihinen K., Kärenlampi S., Hohtola A. (2004). Activation of flavonoid biosynthesis by solar radiation in bilberry (*Vaccinium myrtillus* L) leaves. Planta.

[160] Martineau L.C., Couture A., Spoor D., Benhaddou-Andaloussi A., Harris C., Meddah B., Leduc C., Burt A., Vuong T., Mai Le P. (2006). Anti-diabetic properties of the Canadian lowbush blueberry *Vaccinium angustifolium* Ait. Phytomedicine.

[161] Martz F., Jaakola L., Julkunen-Tiitto R., Stark S. (2010). Phenolic composition and antioxidant capacity of bilberry (*Vaccinium myrtillus*) leaves in Northern Europe following foliar development and along environmental gradients. J. Chem. Ecol..

[162] Helmstädter A., Schuster N. (2010). *Vaccinium myrtillus* as an antidiabetic medicinal plant--research through the ages. Pharmazie.

[163] Piljac-Žegarac J., Stipčević T., Belščak A. (2009). Antioxidant properties and phenolic content of different floral origin honeys. J. ApiProd. ApiMed. Sci..

[164] Kumar J.P., Prasada Rao C.M.M., Singh R.K., Garg A., Rajeswari T. (2024). A brief review on encapsulation of natural poly-phenolic compounds. Adv. Pharm. J..

[165] Bamidele O.P., Emmambux M.N. (2020). Encapsulation of bioactive compounds by “extrusion” technologies: A review. Crit. Rev. Food Sci. Nutr..

[166] Onsaard E., Onsaard W., Salaün F. (2019). Microencapsulated Vegetable Oil Powder. Microencapsulation—Processes, Technologies and Industrial Applications.

[167] Rezagholizade-shirvan A., Soltani M., Shokri S., Radfar R., Arab M., Shamloo E. (2024). Bioactive compound encapsulation: Characteristics, applications in food systems, and implications for human health. Food Chem. X.

[168] Timilsena Y., Haque M., Adhikari B. (2020). Encapsulation in the Food Industry: A Brief Historical Overview to Recent Developments. Food Nutr. Sci..

[169] Vila M.M.D.C., Chaud M.V., Balcão V.M., Sagis L.M.C. (2015). Chapter 19—Microencapsulation of Natural Anti-Oxidant Pigments. Microencapsulation and Microspheres for Food Applications.

[170] Azhar M.D., Hashib S.A., Ibrahim U.K., Rahman N.A. (2021). Development of carrier material for food applications in spray drying technology: An overview. Mater. Today Proc..

[171] Marcillo-Parra V., Tupuna-Yerovi D.S., González Z., Ruales J. (2021). Encapsulation of bioactive compounds from fruit and vegetable by-products for food application—A review. Trends Food Sci. Technol..

[172] Rezvankhah A., Emam-Djomeh Z., Askari G. (2020). Encapsulation and delivery of bioactive compounds using spray and freeze-drying techniques: A review. Dry. Technol..

[173] Ledri S.A., Milani J.M., Shahidi S.A., Golkar A. (2024). Comparative analysis of freeze drying and spray drying methods for encapsulation of chlorophyll with maltodextrin and whey protein isolate. Food Chem. X.

[174] Lu Y.S., Liang X.R., Cheng L.S., Fang S. (2020). Microencapsulation of Pigments by Directly Spray-Drying of Anthocyanins Extracts from Blueberry Pomace: Chemical Characterization and Extraction Modeling. Int. J. Food Eng..

[175] Flores F.P., Singh R.K., Kerr W.L., Pegg R.B., Kong F. (2014). Total phenolics content and antioxidant capacities of microencapsulated blueberry anthocyanins during in vitro digestion. Food Chem..

[176] Bittencourt L.L.D., Silva K.A., de Sousa V.P., Fontes-Sant’Ana G.C., Rocha-Leao M.H. (2018). Blueberry Residue Encapsulation by Ionotropic Gelation. Plant Foods Hum. Nutr..

[177] Tzatsi P., Goula A.M. (2021). Encapsulation of Extract from Unused Chokeberries by Spray Drying, Co-crystallization, and Ionic Gelation. Waste Biomass Valorization.

[178] Eisinaitė V., Leskauskaitė D., Pukalskienė M., Venskutonis P.R. (2020). Freeze-drying of black chokeberry pomace extract-loaded double emulsions to obtain dispersible powders. J. Food Sci..

[179] Cezarotto V.S., Franceschi E.P., Stein A.C., Emanuelli T., Maurer L.H., Sari M.H.M., Ferreira L.M., Cruz L. (2023). Nanoencapsulation of *Vaccinium ashei* Leaf Extract in Eudragit^®^ RS100-Based Nanoparticles Increases Its In Vitro Antioxidant and In Vivo Antidepressant-like Actions. Pharmaceuticals.

[180] Ștefănescu B.E., Nemes S.A., Teleky B.E., Călinoiu L.F., Mitrea L., Martău G.A., Szabo K., Mihai M., Vodnar D.C., Crișan G. (2022). Microencapsulation and Bioaccessibility of Phenolic Compounds of Vaccinium Leaf Extracts. Antioxidants.

[181] Tchabo W., Ma Y., Kaptso G.K., Kwaw E., Cheno R.W., Xiao L., Osae R., Wu M., Farooq M. (2019). Process Analysis of Mulberry (*Morus alba*) Leaf Extract Encapsulation: Effects of Spray Drying Conditions on Bioactive Encapsulated Powder Quality. Food Bioprocess Technol..

[182] Tchabo W., Ma Y., Kaptso G.K., Kwaw E., Cheno R.W., Wu M., Osae R., Ma S., Farooq M. (2018). Carrier Effects on the Chemical and Physical Properties of Freeze-Dried Encapsulated Mulberry Leaf Extract Powder. Acta Chim. Slov..

[183] Păvăloiu R.D., Sha’at F., Neagu G., Deaconu M., Bubueanu C., Albulescu A., Sha’at M., Hlevca C. (2021). Encapsulation of Polyphenols from *Lycium barbarum* Leaves into Liposomes as a Strategy to Improve Their Delivery. Nanomaterials.

[184] Raczkowska E., Nowicka P., Wojdyło A., Styczyńska M., Lazar Z. (2022). Chokeberry Pomace as a Component Shaping the Content of Bioactive Compounds and Nutritional, Health-Promoting (Anti-Diabetic and Antioxidant) and Sensory Properties of Shortcrust Pastries Sweetened with Sucrose and Erythritol. Antioxidants.

[185] Szydłowska M., Wojdyło A., Nowicka P. (2024). Black and Red Currant Pomaces as Raw Materials to Create Smoothies with In Vitro Health-Promoting Potential. Foods.

[186] Kidon M., Marciszak E., Ugur S., Kuligowski M., Radziejewska-Kubzdela E. (2023). Chokeberry Pomace Utilization for Improving Selected Quality Parameters of Green Tea Leaves or Hibiscus Flower Infusions. Appl. Sci..

[187] Rohm H., Brennan C., Turner C., Günther E., Campbell G., Hernando I., Struck S., Kontogiorgos V. (2015). Adding Value to Fruit Processing Waste: Innovative Ways to Incorporate Fibers from Berry Pomace in Baked and Extruded Cereal-based Foods—A SUSFOOD Project. Foods.

[188] Quiles A., Llorca E., Schmidt C., Reissner A.M., Struck S., Rohm H., Hernando I. (2018). Use of berry pomace to replace flour, fat or sugar in cakes. Int. J. Food Sci. Technol..

[189] Zbikowska A., Lukasiak P., Kowalska M., Lukasiak A., Kozlowska M., Marciniak-Lukasiak K. (2024). Incorporation of Chokeberry Pomace into Baked Products: Influence on the Quality of the Dough and the Muffins. Appl. Sci..

[190] Šarić B., Mišan A., Mandić A., Nedeljković N., Pojić M., Pestorić M., Đilas S. (2016). Valorisation of raspberry and blueberry pomace through the formulation of value-added gluten-free cookies. J. Food Sci. Technol..

[191] Bialek M., Rutkowska J., Bialek A., Adamska A. (2016). Oxidative Stability of Lipid Fraction of Cookies Enriched with Chokeberry Polyphenols Extract. Pol. J. Food Nutr. Sci..

[192] Górnas P., Juhnevica-Radenkova K., Radenkovs V., Misina I., Pugajeva I., Soliven A., Seglina D. (2016). The impact of different baking conditions on the stability of the extractable polyphenols in muffins enriched by strawberry, sour cherry, raspberry or black currant pomace. LWT.

[193] Alba K., MacNaughtan W., Laws A.P., Foster T.J., Campbell G.M., Kontogiorgos V. (2018). Fractionation and characterisation of dietary fibre from blackcurrant pomace. Food Hydrocolloid.

[194] Struck S., Straube D., Zahn S., Rohm H. (2018). Interaction of wheat macromolecules and berry pomace in model dough: Rheology and microstructure. J. Food Eng..

[195] Reissner A.M., Beer A., Struck S., Rohm H. (2020). Pre-Hydrated Berry Pomace in Wheat Bread: An Approach Considering Requisite Water in Fiber Enrichment. Foods.

[196] Schmidt C., Geweke I., Struck S., Zahn S., Rohm H. (2018). Blackcurrant pomace from juice processing as partial flour substitute in savoury crackers: Dough characteristics and product properties. Int. J. Food Sci. Technol..

[197] Martins Z.E., Pinho O., Ferreira I. (2017). Fortification of Wheat Bread with Agroindustry By-Products: Statistical Methods for Sensory Preference Evaluation and Correlation with Color and Crumb Structure. J. Food Sci..

[198] Kitchen K. (2007). Polyphenolic-Rich Products Made with Georgia-Grown Rabbiteye Blueberries. Ph.D. Thesis.

[199] Diez-Sánchez E., Quiles A., Llorca E., Reissner A.M., Struck S., Rohm H., Hernando I. (2019). Extruded flour as techno-functional ingredient in muffins with berry pomace. LWT.

[200] Curutchet A., Cozzano S., Tárrega A., Arcia P. (2019). Blueberry pomace as a source of antioxidant fibre in cookies: Consumer’s expectations and critical attributes for developing a new product. Food Sci. Technol. Int..

[201] Reissner A.M., Struck S., Alba K., Proserpio C., Foschino R., Turner C., Hernando I., Zahn S., Rohm H. (2021). Cross-national differences in consumer responses to savoury crackers containing blackcurrant pomace. Int. J. Food Sci. Technol..

[202] Vázquez C.V., Rojas M.G.V., Ramírez C.A., Chávez-Servín J.L., García-Gasca T., Ferriz Martínez R.A., García O.P., Rosado J.L., López-Sabater C.M., Castellote A.I. (2015). Total phenolic compounds in milk from different species. Design of an extraction technique for quantification using the Folin–Ciocalteu method. Food Chem..

[203] Sankowski L.V., Morales-Medina R., Arguello C.F., Reibner A.M., Struck S., Rohm H., Drusch S., Brückner-Gühmann M. (2024). Thermal-mechanical treatment of blackcurrant pomace for enrichment in yoghurt. Food Hydrocolloid.

[204] Du H.X., Yang H.G., Wang X.P., Zhu F., Tang D.B., Cheng J.R., Liu X.M. (2021). Effects of mulberry pomace on physicochemical and textural properties of stirred-type flavored yogurt. J. Dairy Sci..

[205] Raikos V., Ni H., Hayes H., Ranawana V. (2019). Antioxidant Properties of a Yogurt Beverage Enriched with Salal (*Gaultheria shallon*) Berries and Blackcurrant (*Ribes nigrum*) Pomace during Cold Storage. Beverages.

[206] Ni H., Hayes H.E., Stead D., Raikos V. (2018). Incorporating salal berry (*Gaultheria shallon*) and blackcurrant (*Ribes nigrum*) pomace in yogurt for the development of a beverage with antidiabetic properties. Heliyon.

[207] Trajkovska B., Nakov G., Prabhat S.T., Badgujar P.C. (2024). Effect of Blueberry Pomace Addition on Quality Attributes of Buttermilk-Based Fermented Drinks during Cold Storage. Foods.

[208] Cheng Y.X., Wu T., Chu X.Q., Tang S.X., Can W.W., Liang F.Q., Fang Y.J., Pan S.Y., Xu X.Y. (2020). Fermented blueberry pomace with antioxidant properties improves fecal microbiota community structure and short chain fatty acids production in an in vitro mode. LWT.

[209] Silva S., Costa E.M., Mendes M., Morais R.M., Calhau C., Pintado M.M. (2016). Antimicrobial, antiadhesive and antibiofilm activity of an ethanolic, anthocyanin-rich blueberry extract purified by solid phase extraction. J. Appl. Microbiol..

[210] Babaoğlu A.S., Unal K., Dilek N.M., Poçan H.B., Karakaya M. (2022). Antioxidant and antimicrobial effects of blackberry, black chokeberry, blueberry, and red currant pomace extracts on beef patties subject to refrigerated storage. Meat Sci..

[211] Salaheen S., Jaiswal E., Joo J., Peng M.F., Ho R., OConnor D., Adlerz K., Aranda-Espinoza J.H., Biswas D. (2016). Bioactive extracts from berry byproducts on the pathogenicity of *Salmonella* Typhimurium. Int. J. Food Microbiol..

[212] Taraseviciene Z., Cechoviciene I., Paulauskiene A., Gumbyte M., Blinstrubiene A., Burbulis N. (2022). The Effect of Berry Pomace on Quality Changes of Beef Patties during Refrigerated Storage. Foods.

[213] Tamkute L., Vaicekauskaite R., Melero B., Jaime I., Rovira J., Venskutonis P.R. (2021). Effects of chokeberry extract isolated with pressurized ethanol from defatted pomace on oxidative stability, quality and sensory characteristics of pork meat products. LWT.

[214] Sivapragasam N., Maqsood S., Rupasinghe H.P.V. (2024). Berry bioactive compounds immobilized in starch matrix for active and intelligent packaging: A review. Future Foods.

[215] Singh A., Gu Y.X., Castellarin S.D., Kitts D.D., Pratap-Singh A. (2020). Development and Characterization of the Edible Packaging Films Incorporated with Blueberry Pomace. Foods.

[216] Kurek M., Benbettaieb N., Scetar M., Chaudy E., Repajic M., Klepac D., Valic S., Debeaufort F., Galic K. (2021). Characterization of Food Packaging Films with Blackcurrant Fruit Waste as a Source of Antioxidant and Color Sensing Intelligent Material. Molecules.

[217] Tauferova A., Pospiech M., Javurkova Z., Tremlova B., Dordevic D., Jancikova S., Tesikova K., Zdarsky M., Vitez T., Vitezova M. (2021). Plant Byproducts as Part of Edible Coatings: A Case Study with Parsley, Grape and Blueberry Pomace. Polymers.

[218] Yang G., Yue J., Gong X., Qian B., Wang H., Deng Y., Zhao Y. (2014). Blueberry leaf extracts incorporated chitosan coatings for preserving postharvest quality of fresh blueberries. Postharvest Biol. Technol..

[219] Nemetz N.J., Schieber A., Weber F. (2021). Application of Crude Pomace Powder of Chokeberry, Bilberry, and Elderberry as a Coloring Foodstuff. Molecules.

[220] Hasan M.M., Islam M.R., Haque A.R., Kabir M.R., Khushe K.J., Hasan S.M.K. (2024). Trends and challenges of fruit by-products utilization: Insights into safety, sensory, and benefits of the use for the development of innovative healthy food: A review. Bioresour. Bioprocess..

